# Sinapoylglucose: malate sinapoyltransferase SNG1 negatively regulates drought resistance by crosstalk with abscisic acid signaling in Arabidopsis

**DOI:** 10.1111/tpj.71102

**Published:** 2026-09-17

**Authors:** Kun Li, Jiangtao Jia, Yong Yang, Minghui Zhang, Shulin Zhang, Xiaohan Liang, Zhiwen Lu, Min Xu, Zhe Wang, Fengbo Yang, Haipeng Li, Jinggong Guo, Kun‐peng Jia, Lam‐Son Phan Tran, Jose R. Botella, Weiqiang Li, Yuchen Miao

**Affiliations:** ^1^ National Key Laboratory of Cotton Bio‐breeding and Integrated Utilization, Henan Joint International Laboratory for Crop Multi‐Omics Research, School of Life Sciences Henan University Jinming Road Kaifeng 475004 China; ^2^ Sanya Institute of Henan University Sanya Hainan 572025 China; ^3^ Department of Plant and Soil Science, Institute of Genomics for Crop Abiotic Stress Tolerance Texas Tech University Lubbock Texas 79409 USA; ^4^ School of Agriculture and Food Sustainability University of Queensland Brisbane Queensland 4072 Australia; ^5^ Jilin Da'an Agro‐ecosystem National Observation and Research Station, State Key Laboratory of Black Soils Conservation and Utilization, Northeast Institute of Geography and Agroecology Chinese Academy of Sciences Changchun Jilin 130102 China

**Keywords:** metabolomic analysis, abscisic acid, anthocyanin, cuticle, drought, root hair, sinapoylglucose, SNG1

## Abstract

Drought stress has a profound impact on yield, with massive repercussions for agricultural production. In addition to ABA, the central hormone controlling the plant response to drought, secondary metabolites also play important functions in drought stress; however, the precise nature of their roles is still obscure. Metabolomics analysis of ABA‐ and PEG‐treated Arabidopsis wild–type (WT) seedlings as well as ABA signal transduction mutants *snrk‐triple* (*snrk2.2 snrk2.3 snrk2.6*) and *pyl‐quadruple* (*pyr1 pyl1 pyl2 pyl4*) identified a number of drought‐related metabolites. Specifically, we observed differences in the levels of sinapic acid and quercetin. To assess the function of sinapoyl esters in the regulation of drought stress, we characterized the *sinapoylglucose accumulator 1* (*sng1*) mutant, deficient in the conversion of sinapoylglucose (SG) to sinapoylmalate (SM), resulting in high endogenous levels of SG and low SM levels. *sng1* mutants showed increased drought resistance compared to WT plants. RNA‐sequencing of *sng1* and WT rosette leaves before and after dehydration revealed differentially expressed genes related to cuticle synthesis, anthocyanin biosynthesis, leaf senescence and ABA response. Consistently, phenotypic analysis of the *sng1* mutant showed increased cuticle synthesis, wax crystal deposition, anthocyanin accumulation, leaf senescence, ABA responsiveness and stomatal closure compared to WT plants. We propose that the deficiency of SM and accumulation of SG in *sng1* mutants may affect the metabolic flow of sinapoyl esters to activate multiple biological processes whose combined action enhances drought resistance. Our findings shed light on the secondary metabolites regulatory network in response to drought stress in Arabidopsis.

## Significance Statement

Secondary metabolites are vital for ABA‐mediated plant drought tolerance, and characterizing their functions under ABA drought signalling is a core biological research focus. Using metabolomics, this study identifies sinapic acid as a central ABA‐dependent secondary metabolite coordinating Arabidopsis drought responses. *SNG1* modulates cuticle synthesis, anthocyanin biosynthesis, leaf senescence, stomatal closure and root hair development, with SG and its downstream derivatives serving as key functional mediators.

## INTRODUCTION

Plant secondary metabolites are a large group of biologically active substances important for plant growth and responses to multiple stresses (Chen et al., 2022; Yeshi et al., 2022). Although secondary metabolites are not essential for plant survival, they play an essential role in the response to biotic and abiotic stress (Zhan et al., 2022). Drought stress severely affects crop development and yield, leading to massive losses worldwide and the problem is expected to become more serious due to global climate change. Plants' response to drought stress is highly complex as it affects many processes which are regulated by different secondary metabolites, including water relations between plant and soil, nutrient acquisition and assimilation, stomatal movement, photosynthesis, redox homeostasis and hormone signaling (Kim et al., 2024; Muhammad Aslam et al., 2022). According to the different core structures and chemical nature, plant secondary metabolites can be divided into three major categories: terpenoids/isoprenoids, nitrogen‐containing chemical compounds and phenolics (Zhan et al., 2022). Phenolic compounds contain at least one phenol ring and one hydroxyl group and can be divided into four groups: phenolic acids, flavonoids, tannins and stilbenes (Rasouli et al., 2016; Zhan et al., 2022). Phenolics play a positive role in the plant resistance to various stresses and have anti‐oxidant and therapeutic properties. Phenolic levels, including anthocyanins and other flavonoids, are affected by drought stress. Several studies have identified a few key phenolic compounds involved in resistance to drought stress; however, their molecular mechanisms are still unclear (Park et al., 2023; Varela et al., 2016). Abscisic acid (ABA) plays a vital role in plant development and the response to environmental stresses, particularly drought (Singh & Roychoudhury, 2023; Waadt et al., 2022). ABA also plays an important role in the production of anthocyanins during drought stress (González‐Villagra et al., 2019). However, the involvement of other phenolic compounds in ABA‐regulated drought resistance remains unexplored.

The phenylpropanoid pathway is an important branch of plant secondary metabolism that produces hundreds of metabolites involved in growth regulation, development, primary metabolism and responses to abiotic and biotic stresses (Dong & Lin, 2021; Vanholme, De Meester, et al., 2019). These metabolites include both large insoluble and small soluble molecules. The main insoluble phenolic products include lignin and suberin in the plant cell walls that provide structure and support, act as physical barriers to pests and pathogens, and enhance environmental stress responses (Barros et al., 2015; Dong & Lin, 2021; Duran‐Medina et al., 2021; Fraser & Chapple, 2011). Major small soluble phenolic compounds include coumarins, flavonoids, stilbenes, lignans and hydroxycinnamoyl esters and amides (Fraser & Chapple, 2011). Most of these simple phenolics are phenylpropanoid intermediates and end products that function in plant growth, development and stress responses (Barros et al., 2015; Dong & Lin, 2021; Vanholme, El Houari, & Boerjan, 2019). Small soluble phenolics are also thought to act as potent regulators of biological processes (Duran‐Medina et al., 2021; Erb & Kliebenstein, 2020; Vanholme, El Houari, & Boerjan, 2019).

Synthesized by a sub‐branch of the phenylpropanoid metabolism pathway, sinapoyl esters are derived from feruloyl‐CoA through a series of reactions, beginning with methylation of the 3‐hydroxyl of caffeoyl‐CoA by caffeic acid O‐methyltransferase. This is followed by the activities of cinnamoyl‐CoA reductase (CCR), ferulate 5‐hydroxylase (F5H, also called ferulic acid hydroxylase, FAH1), and reduced epidermal fluorescence 1 aldehyde dehydrogenase, which ultimately produce sinapic acid (Fraser & Chapple, 2011). Previous work has shown that there are three main sinapoyl esters in *Arabidopsis thaliana*, sinapoylglucose (SG), sinapoylmalate (SM) and sinapoylcholine (SC) (Fraser & Chapple, 2011; Milkowski et al., 2004). SG is synthesized by linking sinapic acid with glucose in a reaction catalyzed by sinapic acid: uridine diphosphoglucose glucosyltransferase (SGT), and a *SGT*‐deficient mutant with hyper‐fluorescent trichomes named *bright trichomes* (*brt1*) has been described (Sinlapadech et al., 2007). SM is synthesized from SG by SG:malate sinapoyltransferase (SMT) and is present mainly in leaves (Lehfeldt et al., 2000). The *SMT*‐deficient mutant *sinapoylglucose accumulator 1* (*sng1*) contains high levels of SG and undetectable levels of SM in leaves (Lehfeldt et al., 2000). SC is mainly synthesized from SG by SG: choline sinapoyltransferase (SCT, also called SNG2) in developing seeds (Shirley et al., 2001; Shirley & Chapple, 2003); SC is degraded by SC esterase (SCE) to produce sinapic acid and choline during early seed germination (Clauss et al., 2008).

Sinapoyl esters are involved in the regulation of primary and secondary metabolism, growth and development and responses to various stresses (Milkowski & Strack, 2010). Sinapoyl ester deficiency leads to reduced anthocyanin accumulation, as observed in *fah1* and *brt1* mutant plants (Maruta et al., 2014; Anderson et al., 2015). Soluble sinapoyl esters also function as bioactive compounds in early plant developmental stages, regulating seed germination and stomatal opening (Bi et al., 2017; Li et al., 2023). Several reports have suggested that sinapoyl esters can enhance pathogen resistance. For example, *FAH1* and *SNG1* were necessary for resistance to *Botrytis cinerea* infection (Demkura & Ballare, 2012; Kaschani et al., 2009), and *fah1* mutant plants showed impaired resistance to the necrotrophic fungal pathogen *Sclerotinia sclerotiorum* (Huang et al., 2009) and the soil‐borne fungus *Verticillium longisporum* (König et al., 2014). Exogenous application of SG inhibited *V. longisporum* fungal growth *in vitro* (König et al., 2014), indicating that SG was involved in pathogen restriction. In addition, *BRT1* is required for non‐host resistance to *Phakopsora pachyrhizi* in Arabidopsis (Langenbach et al., 2013). These findings indicate that soluble sinapoyl esters are important in the regulation of plant development and pathogen resistance.

The role of sinapoyl esters in resistance to ultraviolet B (UV‐B) radiation was demonstrated in Arabidopsis *fah1* mutants, which were highly susceptible to UV‐B‐induced death (Landry et al., 1995) and inhibition of the photosystem II reaction centre (Booij‐James et al., 2000). Numerous reports have suggested that UV‐B and drought stress provoke similar physiological responses, and UV‐B exposure increases drought resistance in many crops (Poulson et al., 2006). In addition, responses to UV‐B and drought share many common genes, as supported by a recent meta‐analysis of 52 publications documenting their shared physiological and morphological responses, including changes in antioxidant capacity, leaf thickness, height, stomatal movement, chlorophyll content and biomass (Jansen et al., 2022; Saenz‐de la et al., 2021). This evidence suggests that sinapoyl ester metabolism could contribute to drought resistance.

Despite the circumstantial evidence linking sinapoyl metabolism to drought resistance, our understanding of its potential involvement in the drought response is still rudimentary, and whether other secondary metabolites are also involved is still obscure. The aim of this study was to identify secondary metabolites involved in the plant drought response and investigate their roles in drought resistance. Our results show that phenylpropanoid metabolism is affected in seedlings of ABA signal transduction mutants *snrk‐triple* (*snrk2.2 snrk2.3 snrk2.6*) and *pyl‐quadruple* (*pyr1 pyl1 pyl2 pyl4*), and that the phenolic compound is important for drought stress resistance in Arabidopsis. We also show that the *sng1* mutant has increased drought resistance compared to WT plants. Transcriptomic analysis of the *sng1* mutant identified differentially expressed genes related to cuticle synthesis, anthocyanin biosynthesis, leaf senescence and ABA responsiveness, and these findings were supported by physiological measurements. Finally, we observed longer root hairs, higher ABA levels and lower cytokinin levels in the *sng1* mutant compared to WT, indicating that morphological features and hormone levels are affected by SNG1 through the changed levels of sinapoyl esters. Thus, our study suggests that phenylpropanoid metabolism is vital for drought stress resistance in Arabidopsis, and the key enzyme in sinapoyl metabolism, SNG1, negatively regulates drought resistance by affecting multiple physiological, biochemical and signalling processes.

## RESULTS

### Comparative metabolome analyses show that the phenylpropanoid metabolism pathway plays an important role in the Arabidopsis response to drought stress

As a preliminary step to identify secondary metabolites involved in plant drought stress, we measured phenolic metabolite levels in Arabidopsis wild‐type (WT) seedlings treated with 20% PEG6000 using high‐performance liquid chromatography (HPLC). Our results showed that PEG6000 treatment induced the accumulation of phenolic metabolites compared to control samples, with strong differences observed after 9 days of treatment (Figure S1A, red line). In addition, seedling growth was severely inhibited by PEG6000 treatment for 9 days (Figure S1B), with a strong reduction of fresh weight and rosette leaf area (Figure S1C,D). Therefore, treatment with 20% PEG6000 for 9 d was chosen for further experiments.

High‐resolution mass spectrometry (HRMS) analysis of metabolite levels in WT plants under ABA and PEG6000 treatment conditions identified a total of 434 metabolites (Table S1). Principal component analysis (PCA) of these metabolites was conducted to assess the reproducibility of three independent experiments, and two principal components were found to explain 47.6% of the overall variance of metabolite profiles (26.2% and 21.4% for PC1 and PC2, respectively; Figure 2A). In the CK‐vs‐PEG analysis, the levels of 38 metabolites increased by PEG treatment while 18 metabolites showed lower levels. In the CK‐vs‐ABA group, ABA treatment increased endogenous levels of 28 metabolites while reduced levels were detected for another 28 metabolites (Figure 2B). Among the set of metabolites showing differential levels, 39 metabolites belonged to both treatment groups, PEG6000 and ABA (Figure 2C). Metabolic pathway analysis of the 39 common metabolites revealed that they are distributed across 15 metabolic pathways, including glutathione in the glutathione metabolism pathway, quercetin in the flavonoid and flavonoid biosynthesis pathways and sinapate in the phenylpropanoid biosynthesis pathway (Figure 2D,E).

**Figure 1:**
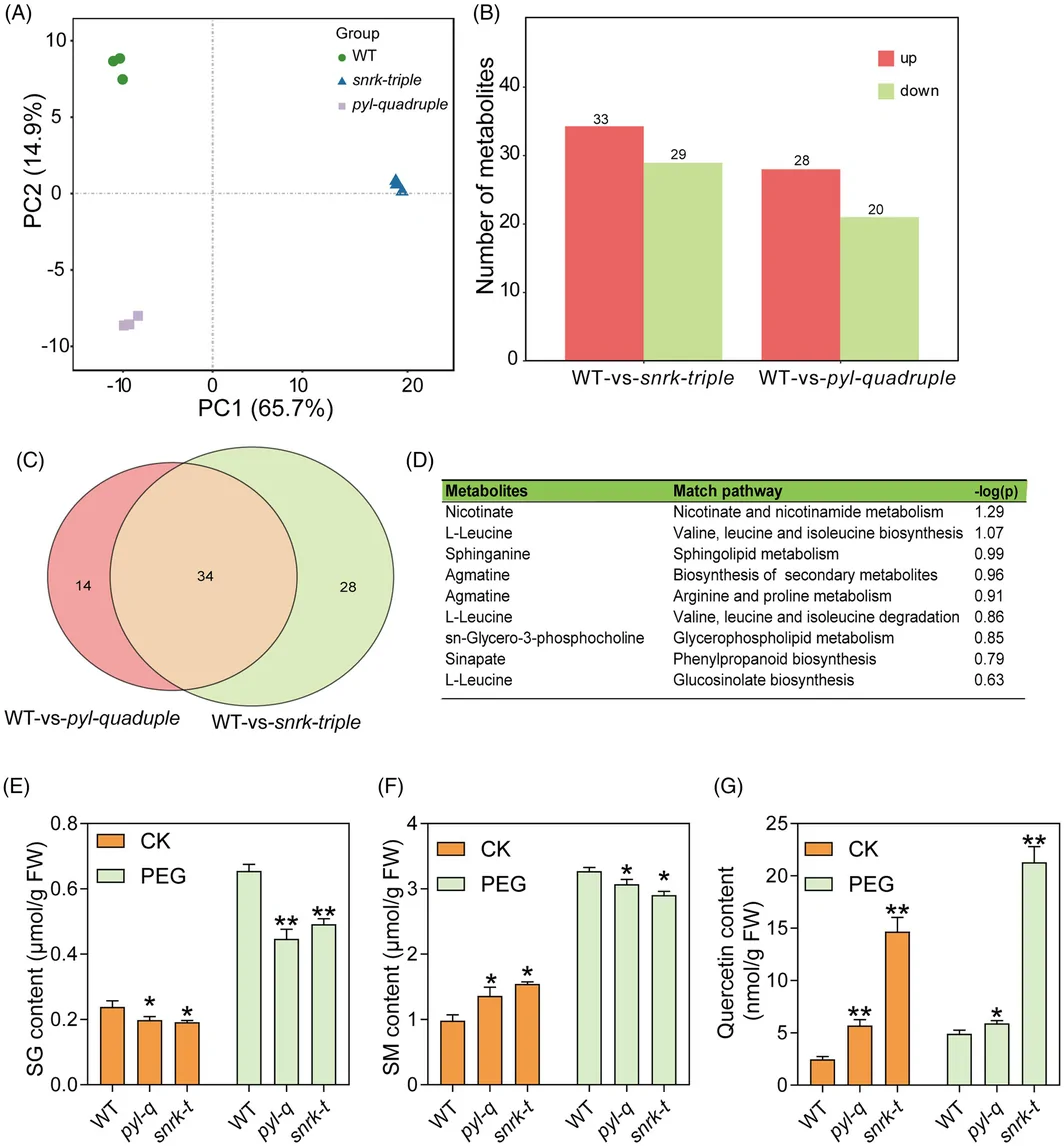
Comparative metabolome analysis of WT, *snrk‐triple* (*snrk2.2 snrk2.3 snrk2.6*, *snrk‐t*) and *pyl‐quadruple* (*pyr1 pyl1 pyl2 pyl4*, *pyl‐q*) mutant plants. (A) Principal component analysis of metabolites from WT, *snrk‐triple* and *pyl‐quadruple* plants. (B) Comparison of metabolites from WT, *snrk‐triple* and *pyl‐quadruple* plants. Red and blue show the high and low content between samples. (C) Venn diagrams showing the common and unique different metabolites from WT, *snrk‐triple* and *pyl‐quadruple* plants. (D) The list of different accumulation metabolites from the common differential metabolites of WT, *snrk‐triple*, and *pyl‐quadruple* plants by pathway impact analysis. (E–G) The content of sinapoylglucose (SG), sinapoylmalate (SM) and quercetin in WT, *snrk‐triple* and *pyl‐quadruple* plants. Data are means and SEs, *n* > 30 plants/genotype under normal growth and PEG treatment conditions. Asterisks indicate significant differences between the genotypes as determined by a Student's *t*‐test, *P < 0.05; **P < 0.01.

We also analysed the ABA signalling pathway mutants, *snrk‐triple* (*snrk2.2 snrk2.3 snrk2.6*) and *pyl‐quadruple* (*pyr1 pyl1 pyl2 pyl4*), identifying a total of 358 metabolites (Table S2). PCA of all these metabolites was conducted to compare their metabolic compositions, and two principal components were found to explain 80.6% of the overall variance of metabolite profiles (65.7% and 14.9% for PC1 and PC2, respectively; Figure 1A). The *snrk‐triple* mutants contained 62 metabolites showing different levels compared to WT controls, while the *pyl‐quadruple mutants* contained 48 differential‐level metabolites (Figure 1B), with 34 of them being common to both mutants (Figure 1C). Pathway analysis of the common 34 metabolites showed that they are distributed across 9 metabolic pathways (Figure 1D). To confirm the metabolome analysis results, the content of sinapic acid and quercetin was measured in WT, *snrk‐triple* and *pyl‐quadruple* plants. Consistent with the metabolome results, we observed a reduction in sinapoylglucose (SG) levels and an increase in sinapoylmalate (SM) in *snrk‐triple* and *pyl‐quadruple* mutants compared to WT controls (Figure 1E,F) in normal growth conditions, and the levels of SG and SM were increased under PEG treatment conditions; the SG and SM contents were still lower than WT controls (Figure 1E,F), while the content of quercetin significantly increased in both mutants (Figure 1G). These results suggest that sinapate and quercetin may have a role in the Arabidopsis response to drought stress.

To clarify whether sinapic acid and quercetin modulate drought tolerance in Arabidopsis, seedlings were separately transferred to culture media containing PEG6000, PEG6000 + sinapic acid, or PEG6000 + quercetin. Under normal growth conditions, exogenous supply of 300 μM sinapic acid or 300 μM quercetin enhanced seedling growth (Figure S3A–C). Different concentrations of PEG6000 treatment substantially retarded seedling development, whereas supplementation with either compound alleviated this PEG6000‐triggered growth repression; moreover, sinapic acid exhibited a significantly stronger mitigating effect on PEG6000‐mediated growth inhibition compared with quercetin (Figure S3A–C). Accordingly, sinapic acid was chosen as the primary research compound in our follow‐up investigations.

### SNG1‐deficient *sng1* mutants are more resistant to drought than WT plants

**Figure 2:**
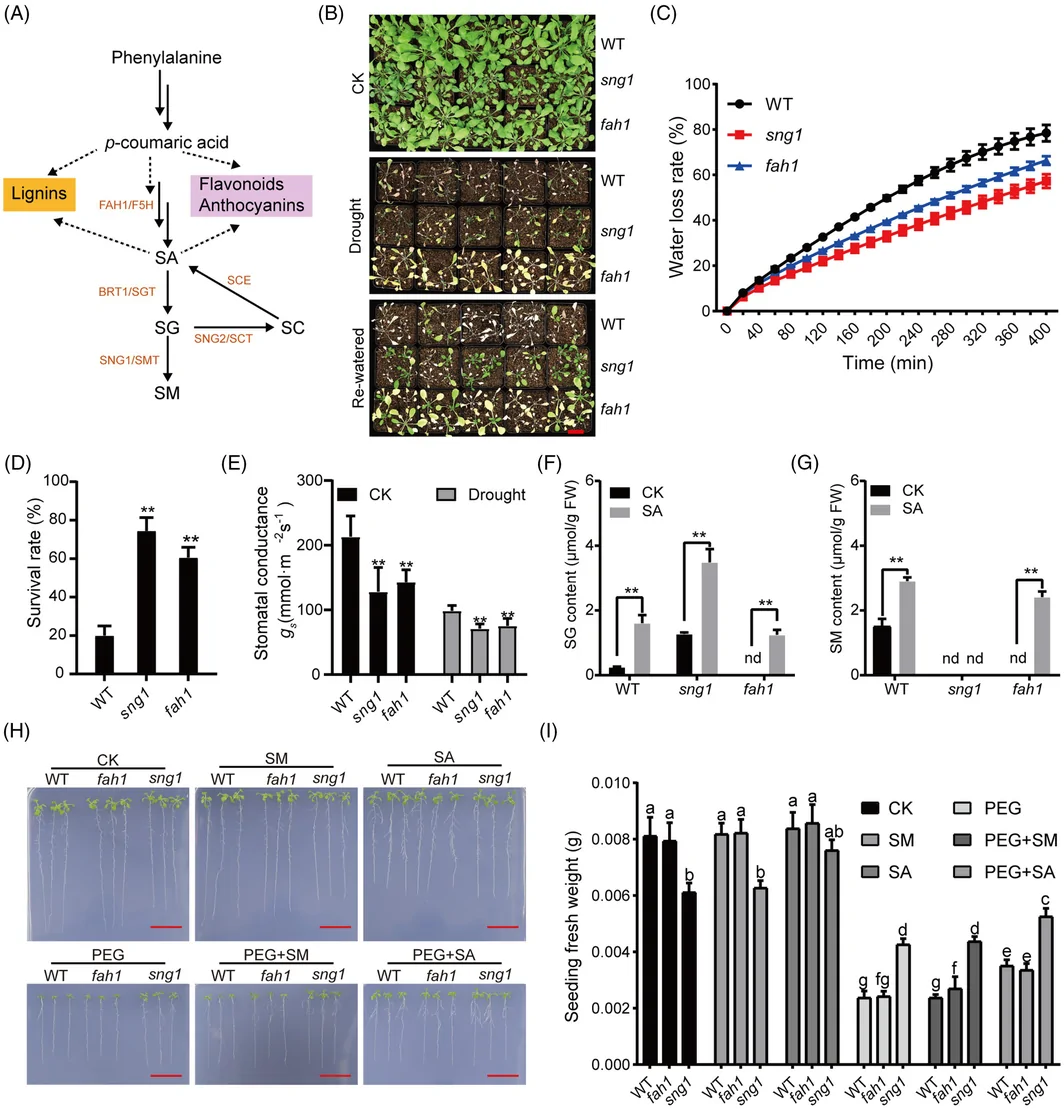
*sng1* and *fah1* plants are more resistant to drought than WT. (A) Schematic diagram of the sinapoyl ester pathway and its crosstalk with lignin and anthocyanin biosynthesis in Arabidopsis. Sinapic acid (SA) is synthesized from *p*‐coumaric acid *via* sequential enzymatic steps in the phenylalanine sub‐branch, with ferulic acid 5‐hydroxylase (F5H/FAH1) as the rate‐limiting enzyme. SA is glucosylated to SG by SGT/BRT1. SG is converted to SM by SMT/SNG1. During seed formation, SG is converted to sinapoylcholine (SC) by SCT/SNG2. SC is hydrolyzed back to SA by SCE during germination. Sinapoyl esters also contribute to lignin and anthocyanin production. Solid arrows: enzymatic steps (enzymes in brown); dashed arrows: branching pathways. (B) Phenotypes of 37‐day‐old seedlings of the WT, *sng1* and *fah1* mutants grown under well‐watered conditions (upper panel); 21‐day‐old plants subjected to drought stress for 16 days (middle panel) and subsequent re‐watering for 3 days (lower panel). Images show representative seedlings per genotype from one experimental replicate. Bar = 5 cm. (C) Analysis of transpirational water loss. 21‐day‐old rosette leaves were detached and allowed to dry at room temperature for the indicated periods. Water loss is presented as a percentage of initial fresh weight. Data represent means ± SD of three biological replicates. (D) Survival rates of WT, *sng1* and *fah1* mutant plants were determined from four independent experiments (data are means and SDs, *n* = 60 plants/genotype/experiment). (E) Stomatal conductance of WT and *sng1* mutant plants under normal and drought conditions was determined from four independent experiments (data are means and SDs, *n* = 10). Asterisks indicate significant differences between the genotypes under normal/drought conditions as determined by a Student's *t*‐test, **P < 0.01. (F, G) The detection of SG and SM contents in 12‐day‐old WT, *sng1* and *fah1* mutants after treatment with 100 μM SA for 1 day. Values are means ± SD of three independent experiments. Asterisks indicate significant changes compared with the CK treatment, as determined by a Student's *t*‐test, **P < 0.01; nd, not detected. (H) Phenotypic analysis of WT, *fah1* and *sng1* mutant Arabidopsis seedlings under control, 300 μM SA, 300 μM SM, PEG6000 (40%), PEG6000 + SA and PEG6000 + SM treatments. Representative images of seedlings after 7 days of treatment. Bars = 2 cm. (I) Fresh weight of seedlings. Data are presented as mean ± SD (*n* = 24). Different letters indicate significant differences among groups (one‐way ANOVA with Tukey's test, *P* < 0.05).

Sinapic acid biosynthesis constitutes a vital sub‐branch of the phenylpropanoid pathway (Figure 2A). Previous research has demonstrated that ferulic acid 5‐hydroxylase (FAH1) catalyzes the conversion of coniferaldehyde to 5‐OH coniferaldehyde; loss of function mutation in *FAH1* affects the biosynthesis of sinapate esters (Anderson et al., 2015), sinapic acid is then conjugated to form SG, SM and sinapoylcholine (SC), while the BRT1/SGT (SA: uridine diphosphoglucose glucosyltransferase) mediates the transformation of SG to SM, and the SNG1/SMT (SG: malate sinapoyltransferase) mediates the transformation of SG to SM (Figure 2A). To assess the role of sinapate in drought resistance, we quantified the transcript levels of *BRT1*, *SNG1* and *FAH1*, and found that *BRT1* and *FAH1* were significantly induced upon PEG6000 treatment for 4 h, while the expression level of *SNG1* was decreased upon PEG6000 treatment for 4 h (Figure S4). Then we characterized the behaviour of Arabidopsis WT, *sng1‐1* (hereafter *sng1*) mutants, *SNG1* overexpressing transgenic lines and *fah1‐2* (hereafter *fah1*) mutants under drought stress conditions. 21‐day‐old WT, *sng1* and *fah1* seedlings grown under normal watering conditions were left unwatered until wilting in leaves and stem bases was apparent in more than half of the seedlings from a genotype (~ 16 days) (Figure 2B). Leaf water loss assays showed that *fah1* mutants displayed slower water loss than WT, and *sng1* mutants lost water more slowly than *fah1* (Figure 2C). Trays were then re‐watered, and 3 days later the survival rates of all the genotypes were assessed, and the well‐watered plants were used as a control. Under this water stress treatment, WT plants exhibited a significantly lower survival rate, 3.7‐fold and 3.1‐fold compared to *sng1* and *fah1* mutants, respectively (Figure 2D), even though the soil moisture of WT and *sng1* plants in the same tray was similar (Figure S5). Under well‐watered conditions, the developmental phenotypes of the three genotypes WT, *sng1* and *OE SNG1* were similar (Figure S6A,B). In contrast, *SNG1* overexpressing lines showed decreased resistance to drought stress compared with WT plants (Figure S6B,C). Stomatal conductance of WT, *sng1* and *fah1* plants under normal and drought conditions was measured, and it was found that *sng1* and *fah1* exhibited lower stomatal conductance compared with WT under well‐watered conditions; drought treatment reduced stomatal conductance in all plants, and *sng1* and *fah1* still maintained a lower stomatal conductance than WT (Figure 2E). Furthermore, we found that the loss‐of‐function mutation of *SNG1* resulted in smaller plant size (Figure S7A) and reduced fresh weight (Figure S7B) compared with WT and *fah1* plants, whereas the maximum quantum yield of PSII photochemistry (Fv/Fm) remained unaffected (Figure S7C,D).

Quantification of SG and SM levels in WT, *sng1* and *SNG1* overexpressing plants showed that SG levels in *sng1* plants were higher than in WT plants under normal growth conditions, while drought stress induced SG accumulation in WT, *sng1* and *SNG1* overexpressing plants (Figure S6D). There was nearly no detectable SM in *sng1* mutants, and drought treatment decreased the normal SM levels in WT and *SNG1* overexpressing plants (Figure S6E). Exogenous application of sinapic acid results in a significant increase in SG content in WT, *sng1* and *fah1* seedlings, and SM content in WT and *fah1* plants (Figure 2F–G). To further investigate the effects of SA on regulating drought tolerance, 5‐day‐old Arabidopsis seedlings were grown for 7 days on media containing PEG6000, PEG6000 + SA or PEG6000 + SM. It was shown that only SA relieved PEG6000‐mediated growth repression (Figure 2H), as evidenced by significantly elevated fresh weight in seedlings supplied with SA (Figure 2I). This finding implies that SG may contribute to mitigating PEG6000‐induced growth repression in Arabidopsis.

Since sinapic acid is one of the precursors for lignin synthesis, in order to test whether the enhanced drought resistance in *sng1* mutants is related to the changed content of sinapoylmalate/sinapoylglucose or is indirect *via* alterations to plant water transport and hydraulics caused by altered lignin composition, we sectioned the stems of WT, *sng1* and *fah1* plants and quantified the content and checked deposition of lignin in xylem based on the autofluorescence under UV light and by phloroglucinol‐HCl staining. No obvious difference in lignin deposition was observed in stems of WT and *sng1* mutant plants, whereas the xylem width of stems from *fah1* mutant plants was much thinner compared to WT and *sng1* mutant plants, indicated by autofluorescence under UV illumination and the colour stained by phloroglucinol‐HCl (Figure S8A). Moreover, the composition of major lignin monomer units was measured in stems of WT, *sng1* and *fah1* plants. Under normal growth conditions, the content of G lignin units was similar among WT, *sng1* and *fah1* plants, while S lignin units were similar in WT and *sng1* mutant plants; no S lignin units were detected in *fah1* mutant plants (Figure S8B). The amount of both G‐ and S‐lignin units was increased to a similar amount under drought conditions in WT, *sng1* and *fah1* mutant plants, except that no S‐lignin units were detected in *fah1* mutant plants (Figure S8B). These results indicate that *sng1* mutants are more resistant to drought stress and suggest that *SNG1* negatively regulates drought resistance *via* the altered content of sinapoylmalate/sinapoylglucose.

### Transcriptome analysis of *sng1* and WT rosette leaves under well‐watered and water stress conditions

**Figure 3:**
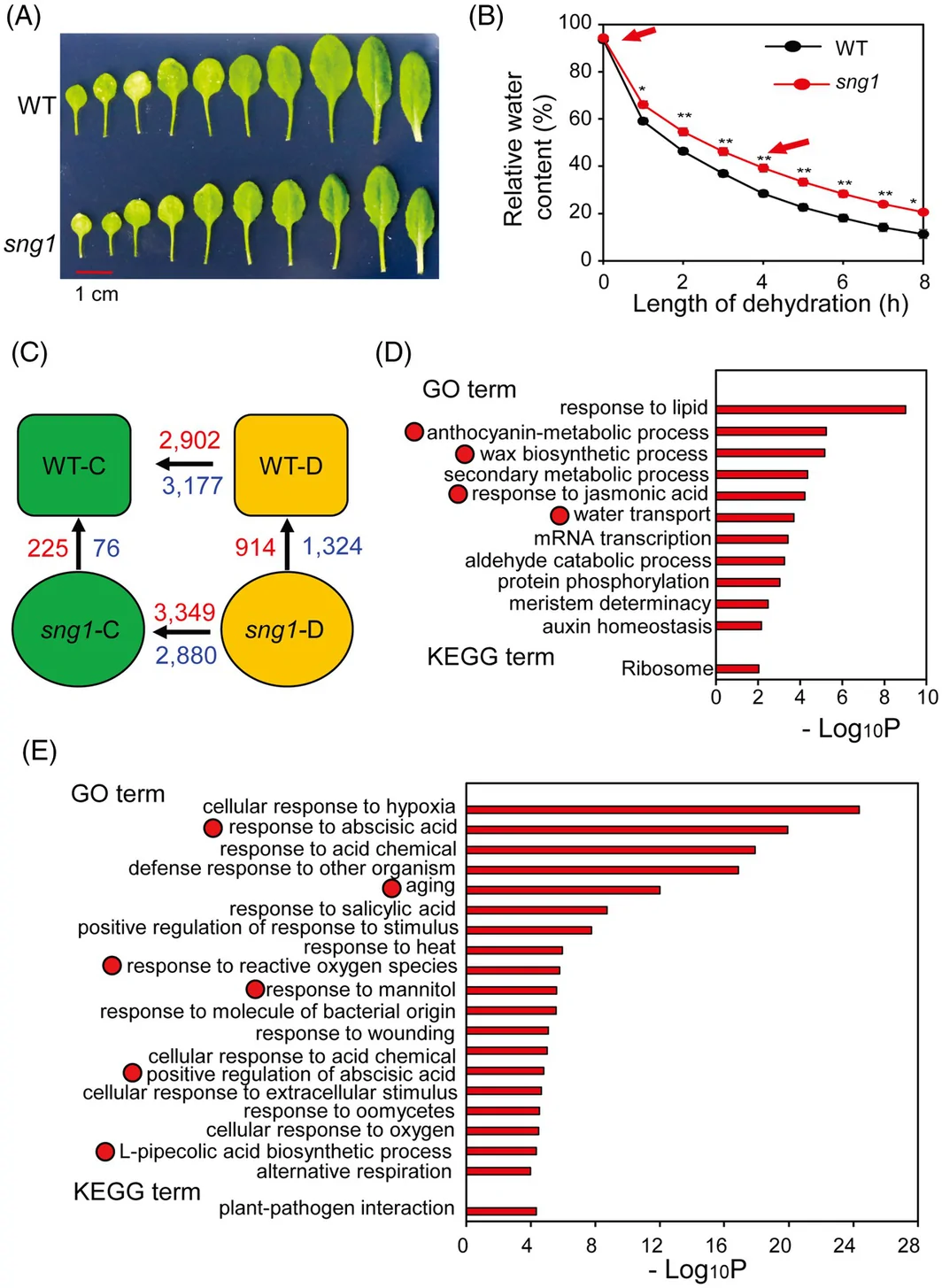
Transcriptome analysis of WT and *sng1* plants under well‐watered and dehydration conditions. (A) Rosette leaves from 24‐day‐old WT and *sng1* plants. (B) Relative water content of rosette leaves from 24‐day‐old WT and *sng1* plants during the dehydration process (data are means and SEs, *n* = 5 plants/genotype). Red arrows indicate the time points of sampling for RNA extraction. Asterisks indicate significant differences between the genotypes under drought conditions as determined by a Student's *t*‐test, *P < 0.05; **P < 0.01. (C) Numbers of differentially expressed genes (DEGs) in comparing different genotype plants under well‐watered and dehydration. Red and blue numbers indicate up‐ and downregulated DEGs, respectively. (D, E) Enriched terms in upregulated DEGs from the ‘*sng1*‐C vs. WT‐C’ (D) and ‘*sng1*‐D vs. WT‐D’ (E) comparisons. Enrichment analysis of Gene Ontology (GO) biological process terms and Kyoto Encyclopedia of Genes and Genomes (KEGG) pathways was performed with Metascape. The x‐axis shows the—log_10_ cumulative hypergeometric *P*‐values of the genes mapped by the GO or KEGG terms and indicates their abundance. The y‐axis shows the enriched GO and KEGG terms. ‘*sng1*‐C vs. WT‐C’, *sng1*well‐watered control versus WT well‐watered control; ‘WT‐D vs. WT‐C’, WT dehydrated 4 h versus WT well‐watered control; ‘*sng1*‐D vs. *sng1*‐C’, *sng1*dehydrated 4 h versus *sng1*well‐watered control; ‘*sng1*‐D vs. WT‐D’, *sng1*dehydrated 4 h versus WT dehydrated 4 h. Red balls indicate those terms are related to drought resistance.

To help elucidate the nature of SNG1 involvement in drought stress, we performed transcriptome analysis of WT and *sng1* rosette leaves under well‐watered and dehydration conditions using RNA‐seq. Rosette leaves from 24‐day‐old WT and *sng1* seedlings were harvested and dehydrated for up to 8 h under lab conditions (Figure 3A,B). Leaf relative water content (RWC) in the *sng1* mutant was significantly higher than that of the WT during dehydration (Figure 3B). Samples for RNA‐Seq were collected at 0 h (WT‐C and *sng1*‐C) and 4 h (WT‐D and *sng1*‐D) after dehydration. Fragments per kilobase per million mapped reads (FPKM) values for all genes in these samples are provided in Table S3, and the original RNA‐seq data are available in NCBI under accession number PRJNA895105. To examine the patterns in transcriptome change, we identified differentially expressed genes (DEGs) in different pairwise comparisons using the criteria |log_2_(fold‐change)| >1 and false discovery rate‐adjusted *P*‐value (i.e. *q*‐value) < 0.05 (Table S4). Under control conditions (0 h), we identified 225 upregulated and 76 downregulated DEGs in *sng1* compared to WT. After dehydration for 4 h, *sng1* leaves had 914 upregulated and 1324 downregulated DEGs compared to WT (Figure 3C, Table S4). We also identified 2902 upregulated and 3177 downregulated DEGs in WT leaves under dehydration conditions, while in *sng1* leaves dehydration resulted in 3349 upregulated and 2880 downregulated DEGs (Figure 3C, Table S4). To validate the RNA‐seq results, several major genes related to drought‐responsive pathways, such as cuticle formation, anthocyanin biosynthesis, ABA metabolism and response, and stomatal movement and senescence, were selected from the DEGs identified by comparing the transcriptomes of WT and *sng1* mutant plants. The expression of these 20 genes was measured by qRT‐PCR, which shows similar expression patterns obtained by RNA‐seq (Figure S9).

### Expression of several drought resistance‐related genes is enhanced in the *sng1* mutant

To gain further insight into SNG1's role in drought resistance, we annotated the DEGs from the ‘*sng1*‐C vs. WT‐C’ and ‘*sng1*‐D vs. WT‐D’ comparisons with Gene Ontology (GO) terms and the Kyoto Encyclopedia of Genes and Genomes (KEGG) pathways and performed enrichment analysis using Metascape software (http://metascape.org) (Zhou et al., 2019). Analysis of upregulated DEGs from the ‘*sng1*‐C vs. WT‐C’ comparison showed enrichment in four drought resistance‐related GO terms: ‘anthocyanin‐metabolic process’, ‘wax biosynthetic process’, ‘response to jasmonic acid’, and ‘water transport’ (Figure 3D, Table S5A). GO terms of upregulated DEGs from the ‘*sng1*‐D vs. WT‐D’ comparison were enriched in six drought‐resistance‐related terms: ‘response to abscisic acid’, ‘ageing’, ‘response to reactive oxygen species’ (ROS), ‘response to mannitol’, ‘positive regulation of abscisic acid’ and ‘L‐pipecolic acid biosynthetic process’ (Figure 3E, Table S5B). These results suggested that the *sng1* mutant may exhibit changes in the regulation of the ABA and JA signalling pathways, secondary metabolic processes (wax, anthocyanin and pipecolic acid metabolism) and ROS homeostasis under normal and/or dehydration conditions.

### Alterations in cuticle and stomata opening contribute to the reduced water loss in *sng1* mutants

**Figure 4:**
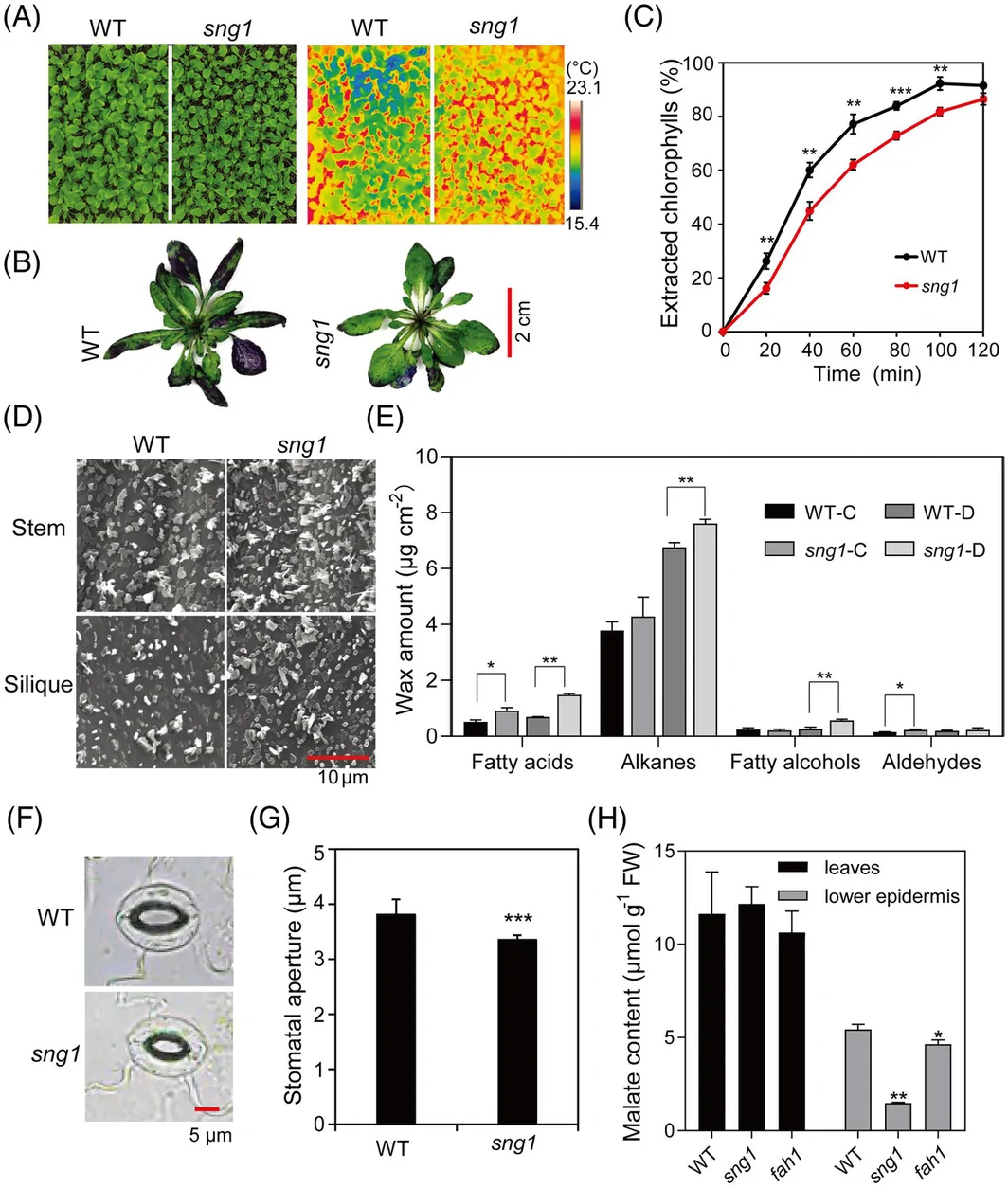
Cuticle formation and wax accumulation are enhanced in *sng1* plants under normal growth conditions. (A) Leaf surface temperatures of WT and *sng1* plants under well‐water conditions. (B) Rosette leaves from 24‐day‐old WT and *sng1* plants after staining with toluidine blue for 4 h, which were grown under well‐watered conditions and indicated the cuticular permeability. (C) Percentage of extracted chlorophyll from the rosette leaves of 24‐day‐old WT and *sng1 mutant* plants grown under well‐watered conditions (data are means and SEs, *n* = 5 plants/genotype). Asterisks indicate significant differences between the genotypes under drought conditions as determined by a Student's *t*‐test, **P < 0.01; ***P < 0.001. (D) Pictures of scanning electron microscopy from stem (*Up*) and silique (*Down*) surfaces of 28‐day‐old soil‐grown plants, which indicated the epicuticular wax crystal deposition in those tissues. (E) Wax amount per unit area in leaves of WT and *sng1* mutant under well‐watered control conditions (C, Control Check) and drought stress conditions (D, Drought). Data are means and SDs (*n* = 3). Asterisks indicate significant differences between the genotypes as determined by a Student's *t*‐test, *P < 0.05, **P < 0.01. (F) Represent pictures of stomata from WT and *sng1* plants. (G) Sizes of stomatal apertures from WT and *sng1* plants. Data are means and SEs (*n* = 5 leaves/genotype, 10 stomata were used from one leaf). Asterisks indicate significant differences between the genotypes as determined by a Student's *t*‐test, ***P < 0.001. (H) Malate content in leaves and lower epidermis of 24‐day‐old WT, *sng1* and *fah1* mutants. Data are means and SDs (*n* = 3). Asterisks indicate significant differences between the genotypes as determined by a Student's *t*‐test, *P < 0.05, **P < 0.01.

Since the ‘wax biosynthetic process’ term was enriched in the ‘*sng1*‐C/WT‐C’ comparison (Figure 3D), we queried whether cuticle synthesis and wax deposition are altered in *sng1* mutants, as enhancement of these processes can reduce leaf water loss through a non‐stomatal mechanism. As an indirect measure of water loss, we measured the leaf surface temperature and observed increased leaf surface temperature in *sng1* mutants compared to WT under well‐watered and dehydration conditions (Figure 4A, Figure S10). To investigate cuticle synthesis, we assessed the cuticular permeability of *sng1* and WT rosette leaves by toluidine blue (TB) staining and chlorophyll (Chl) leaching assays. Consistent with enhanced cuticle synthesis, rosette leaves of *sng1* exhibited reduced staining compared to WT after 4 h of TB staining (Figure 4B), while the rate of Chl leaching was also significantly slower in the *sng1* mutant (Figure 4C).

We hypothesized that increased cuticle synthesis in *sng1* mutants might be related to wax accumulation, as wax content in the cuticle tends to be positively correlated with cuticular water permeability (Cheng et al., 2019; Yeats & Rose, 2013). Scanning electron microscopy (SEM) observations of epicuticular wax crystals on the surfaces of stems and siliques, where wax is preferentially deposited, revealed increased amounts of wax crystals in the *sng1* mutant compared to WT under well‐watered conditions (Figure 4D), suggesting that SNG1 inhibits wax biosynthesis and cuticle formation. Moreover, leaves wax content and composition of WT and *sng1* mutant were measured by GC–MS under normal/drought conditions, and the data showed that the mutation of *SNG1* resulted increased content of fatty acids and aldehydes than WT plants, the amounts of fatty acids, alkanes and fatty alcohols were increased in both WT and *sng1* mutant plants under drought stress, with higher amount in *sng1* mutant than that of WT plants (Figure 4E). It is consistent with the increased amounts of wax crystals in the *sng1* mutant compared to WT. The increased fatty acids, alkanes and fatty alcohols content in *sng1* mutant leaves wax contrasts with the results of enhanced drought resistance of *sng1* mutant plants. We also found that stomatal apertures in *sng1* mutants were smaller than those of WT plants (Figure 4F–G), suggesting that SNG1 also affects stomatal opening. Malate concentration in guard cells exerts a profound influence on stomatal aperture (Allaway, 1973; Dong et al., 2018; Mimata et al., 2022), to test whether the changed stomatal apertures was related to malate levels in guard cells, the malate content was quantified by UPLC‐MS/MS in WT, *sng1* and *fah1* leaves and leaf abaxial epidermis, the data showed that the same levels of malate in leaves of WT, *sng1* and *fah1*, while malate content in leaf abaxial epidermis of WT was higher than *sng1* and *fah1* (Figure 4H). Taken together, these results suggest that *SNG1* expression increases leaf water loss by promoting stomatal opening and inhibiting cuticle synthesis.

### Leaf senescence and water stress‐related anthocyanins accumulation are enhanced in *sng1* mutants

**Figure 5:**
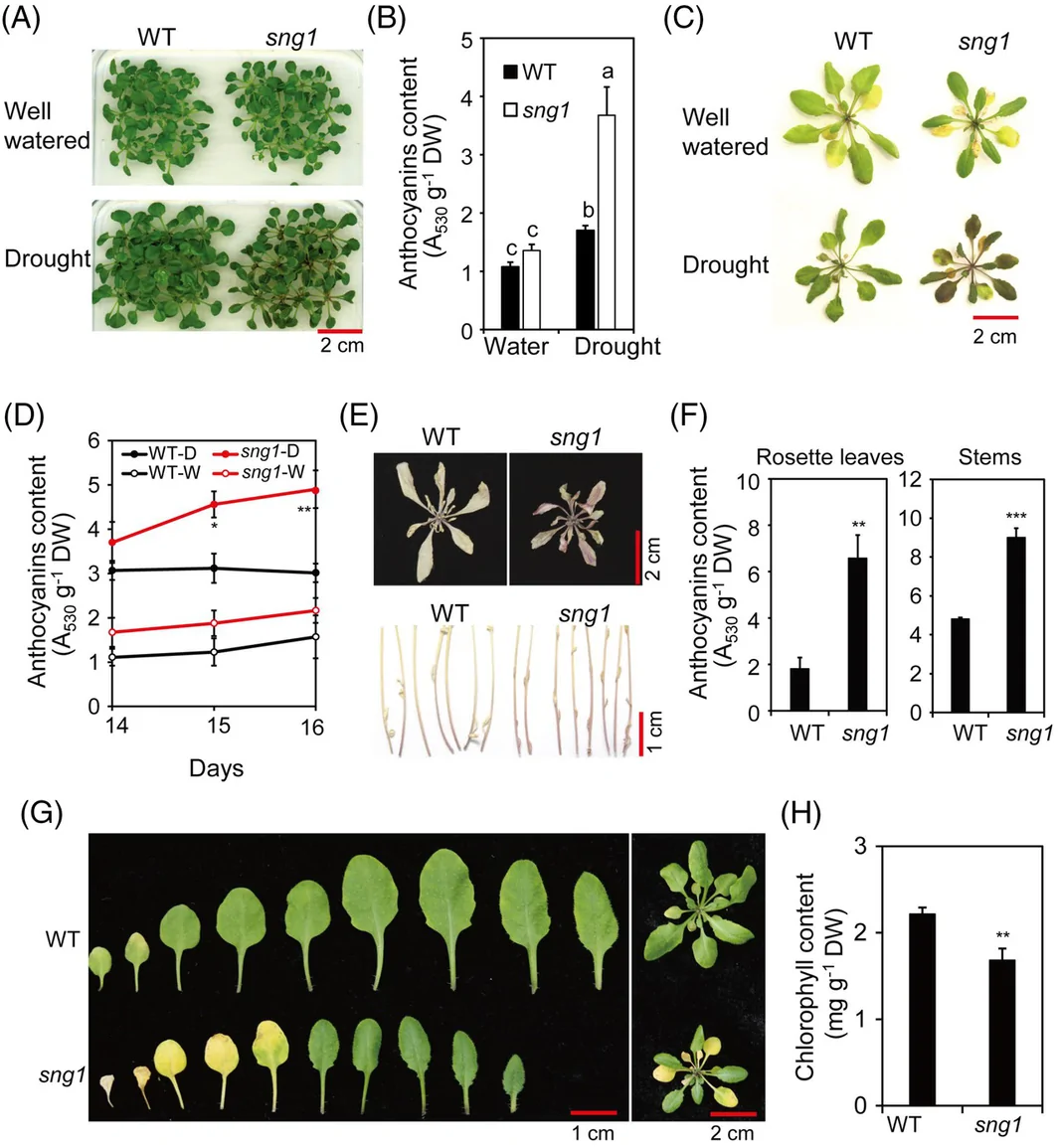
Anthocyanin accumulation and senescence are enhanced in *sng1* plants under well‐watered and drought stress conditions. (A) Plants of *sng1* and WT were grown on 0.5× MS medium for 14 days, then exposed to moving air or not (control) in a clean bench for 1 h and grown in a growth chamber for 4 days. (B) Anthocyanin levels from rosette leaves of (A). Data represent the means and standard errors (*n* = 8). Different letters above the error bars indicate statistically significant differences (*P* < 0.05; ANOVA followed by Tukey's honestly significant difference test) among different genotypes and treatments. (C) Rosette leaves of 36‐day‐old soil–grown *sng1* and WT plants after stopping water for 15 days (drought) and normal well‐watered conditions. (D) Anthocyanin levels from rosette leaves of (C). (E) Rosette leaves and stems of 21‐day‐old soil–grown *sng1* and WT plants after stopping water for 30 days. (F) Anthocyanin levels from rosette leaves and base stems (2 cm from base) of (E). Data represent the means and standard errors (*n* = 7). Asterisks indicate significant differences between the genotypes under drought conditions as determined by a Student's *t*‐test, *P < 0.05; **P < 0.01; ***P < 0.001. (G) Rosette leaves of 31‐day‐old seedlings grown under well‐watered conditions. (H) Chlorophyll contents of rosette leaves of (G). Asterisks indicate significant differences in chlorophyll content between *sng1* and WT determined by Student‘s *t*‐test, ** *P* < 0.01.

Since the ‘anthocyanin metabolic process’ term was enriched in the transcriptomic analysis, and anthocyanins positively contribute to drought resistance (Nakabayashi et al., 2014), we investigated whether drought enhanced anthocyanins accumulation in *sng1* mutants. Analysis of anthocyanins content in agar‐grown *sng1* and WT seedlings under normal conditions revealed similar levels of anthocyanins in *sng1* and WT seedlings (Figure 5A,B). Both genotypes showed increased anthocyanins levels 4 days after exposing the plants to dehydration by opening the agar plates for 1 h, but levels in *sng1* mutants were >2 times higher than those observed in WT seedlings, with a characteristic purple colour clearly visible in the stem bases of *sng1* plants (Figure 5A,B, Figure S11A). To confirm this result, we grew *sng1* and WT plants in soil under normal well‐watered conditions before withholding water for 15–16 d. Consistent with the agar experiments, *sng1* mutant leaves exhibited higher anthocyanins accumulation than WT leaves under water stress conditions (Figure 5C,D, Figure S11B). Interestingly, anthocyanins levels were maintained for a long time, even when the green colour had disappeared 2 weeks after plant death (Figure 5E,F, Figure S11C).

Another of the enriched terms in our transcriptomics analysis was ‘ageing’, and senescence can have a positive effect on drought resistance (Munne‐Bosch & Alegre, 2004; Zhao et al., 2016), we investigated whether leaf senescence was enhanced in *sng1* mutants. Consistent with the transcriptome results, *sng1* plants showed increased senescence (death of old leaves) compared to WT plants under well‐watered and drought stress conditions (Figure 5C,G–H; Figure S11D). Taken together, these results suggest that SNG1 negatively regulates anthocyanin accumulation and leaf senescence under drought stress conditions.

### ABA sensitivity is enhanced in *sng1* mutants

**Figure 6:**
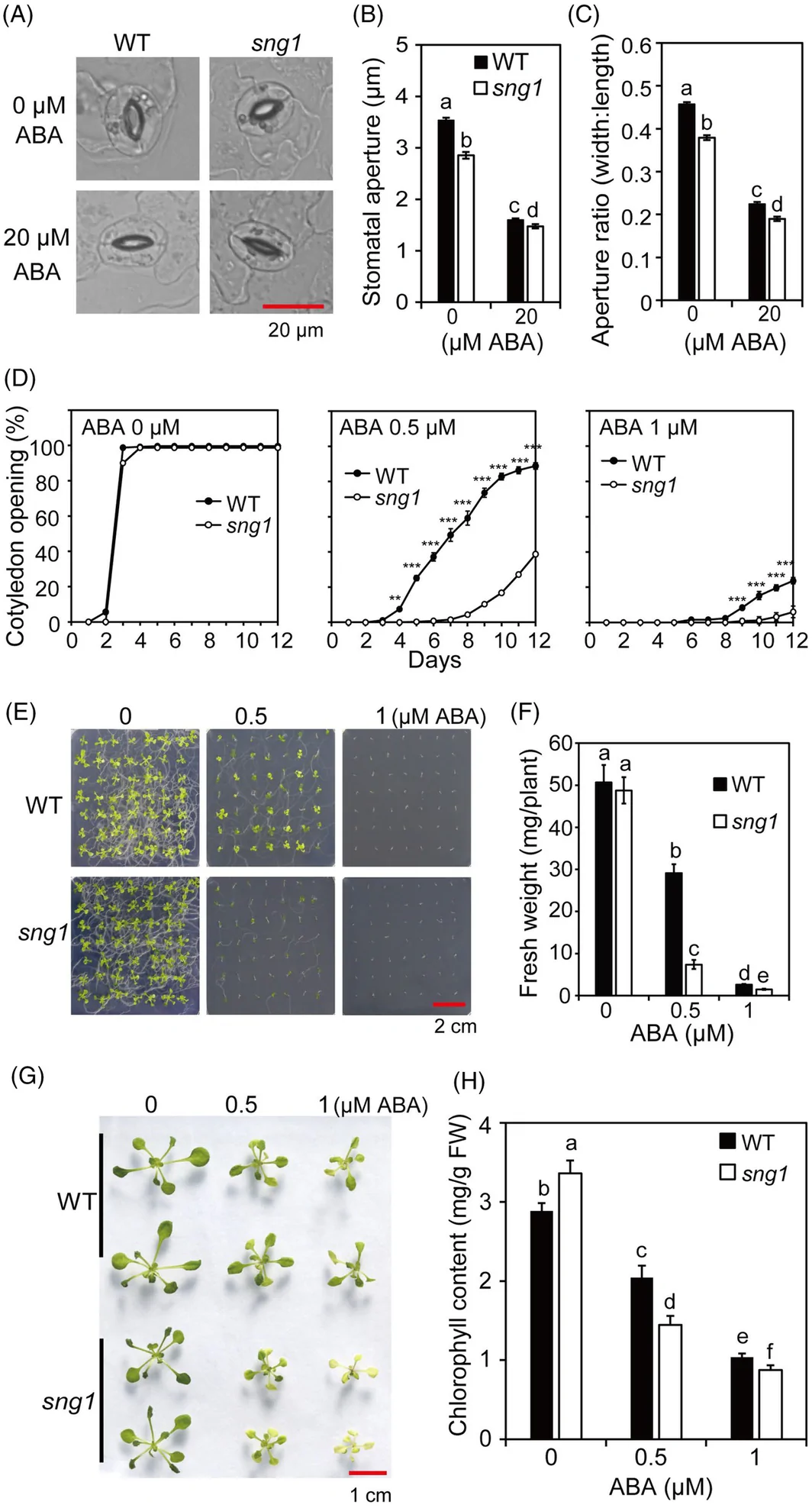
Responsiveness to abscisic acid (ABA) is enhanced in *sng1* plants in cotyledon and seeding growth. (A–C) Pictures (A), stomatal aperture sizes (B) and aperture ratios (C) of WT and *sng1* mutant under different concentrations of ABA. Data are means and SE (*n* = 180). Different letters above the error bars indicate statistically significant differences (*P* < 0.05; ANOVA followed by Tukey's honestly significant difference test) among different genotypes and treatments. (D) Per cent open cotyledons of WT and *sng1* seeds grown under different concentrations of ABA for 0 to 12 days. Data are means and SE of three independent experiments (*n* = 3, 50 seeds/genotype/experiment). Asterisks indicate significant differences between the genotypes under drought conditions as determined by a Student's *t*‐test, **P < 0.01; ***P < 0.001. (E) Seedlings of WT and *sng1* mutant grown in agar with different concentrations of ABA for 12 days. (F) Fresh weights of WT and *sng1* seedlings in (E). (*n* = 5, 6 seedlings/genotype/treatment). Different letters above the error bars indicate statistically significant differences (*P* < 0.05; ANOVA followed by Tukey's honestly significant difference test) among different genotypes and treatments. (G) 10‐day‐old agar–grown seedlings were grown in agar plates containing 0, 0.5, 1 and 2 μM ABA and grown for 12 days. (H) Chlorophyll levels of the shoot in WT and *sng1* plants of (G). (*n* = 4, one seedlings/genotype/treatment). Different letters above the error bars indicate statistically significant differences (*P* < 0.05; ANOVA followed by Tukey's honestly significant difference test) among different genotypes and treatments. According to the bars = 5 μm and 20 μm in Figures 3E and 5A, respectively, the stomatal size seems different.

Our previous research has shown the interaction between ABA signalling and sinapate metabolism during seed germination (Bi et al., 2017), while ABA involvement in drought stress is well characterized (Hsu et al., 2021), and our transcriptomics results showed ABA‐related enrichment terms. We therefore hypothesized that ABA response might be altered in *sng1* mutants. To test our hypothesis, we first measured changes in stomatal aperture size and aperture ratio in response to exogenous ABA application in WT and *sng1* plants. Our results show increased ABA‐induced stomatal closure in *sng1* mutants compared to WT (Figure 6A–C), suggesting increased ABA sensitivity in the *sng1* mutants. Furthermore, we examined cotyledon opening, seedling growth and Chl degradation in *sng1* and WT plants in response to different concentrations of ABA. Exogenous ABA application significantly inhibited cotyledon opening in *sng1* and WT seedlings, but this effect was more pronounced in *sng1* mutants (Figure 6D). Similar results were observed for seedling growth, with increased ABA growth inhibition observed in the *sng1* mutants (Figure 6E,F). ABA‐induced senescence was assayed by quantifying Chl levels after exogenous application of different ABA concentrations. Leaves of the *sng1* mutant contained higher levels of Chl than WT in the absence of ABA (Figure 6G,H). However, addition of 0.5 or 1 μM ABA to the growth medium reduced Chl levels in *sng1* leaves below WT levels (Figure 6H). Similar results were observed when detached leaves were incubated in a 100 μM ABA solution for 2 d (Figure S12A,B). We also found higher endogenous ABA levels and lower endogenous cytokinin levels (*trans*‐zeatin [*t*Z] and *t*Z‐riboside [*t*ZR]) in rosette leaves of the *sng1* mutant compared to WT under well‐watered and drought stress conditions (Figure S12C–E). In summary, these findings indicate that *sng1* mutants contain increased ABA and reduced cytokinin levels compared to WT and demonstrate enhanced ABA response in the *sng1* mutants, suggesting that regulation of these two hormone pathways may account for the increased drought resistance of *sng1* mutants. In addition, qRT‐PCR analysis was performed to detect expression changes of ABA biosynthesis (*NCED3*), catabolism (*BG1*) and drought response genes (*RD29B*, *LEA*) (Zhou et al., 2024) in seedlings sprayed with SA for 2 days. *NCED3* was strongly induced, and *BG1* suppressed, while the drought response genes *RD29B* and *LEA* exhibited remarkably reduced transcript abundance (Figure S12F–I).

### Root hair development is enhanced in *sng1* mutants

**Figure 7:**
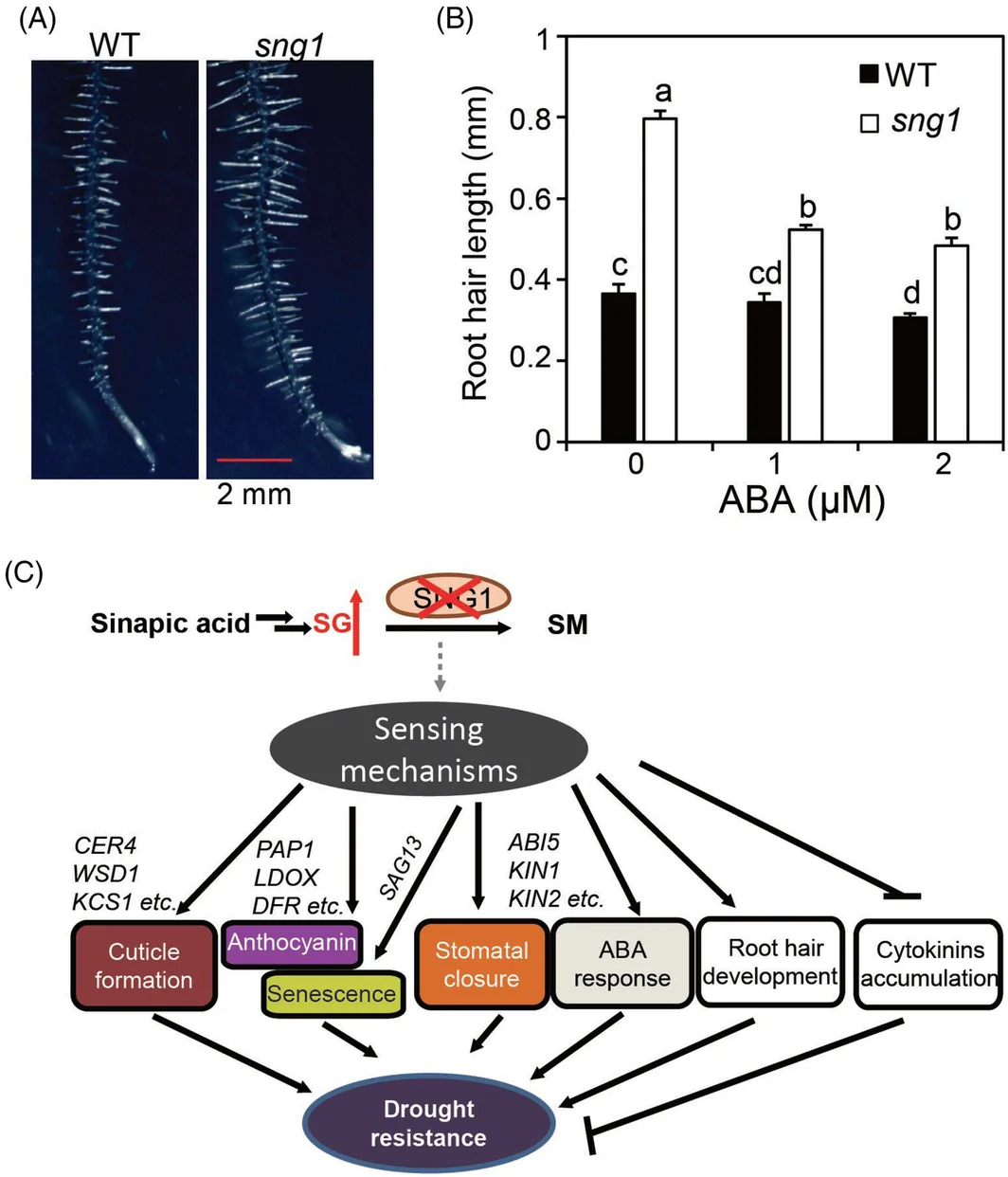
Root hair development is enhanced in *sng1* plants, and a proposed model linking SG accumulation to increased drought resistance. (A) Root hairs of 7‐day‐old WT and *sng1* mutant plants grown in agar. (B) Root hair length of 7‐day‐old WT and *sng1* mutant plants grown in agar with 0, 1 or 2 μM ABA. Different letters above the error bars indicate statistically significant differences (*P* < 0.05; ANOVA followed by Tukey's honestly significant difference test) among different genotypes and treatments. (C) A proposed model showing the negative role of SNG1 in drought resistance. Knockout of *SNG1* leads to SG accumulation, which promotes drought resistance by enhancing cuticle synthesis, anthocyanin accumulation, senescence, stomatal closure, ABA responses and root hair development. Black arrows indicate a promotion effect, and the red arrow indicates accumulation. The grey dotted arrow indicates a hypothesized signal transduction pathway through unknown sensing mechanisms or components.

In addition to the leaf phenotypes described above, we also found significantly longer root hairs and higher root hair density in the *sng1* mutant compared with WT grown in normal agar medium (Figure 7A,B, Figure S13A), suggesting that the *sng1* mutant has increased capacity for water and nutrient absorption from the soil. Addition of 1 μM ABA to the growth medium significantly inhibited root hair elongation in *sng1* but not in WT plants (Figure 7B, Figure S13B), indicating that *sng1* plants were more sensitive to ABA‐induced root hair inhibition. Application of 2 μM ABA significantly inhibited root hair elongation in both *sng1* and WT plants (Figure 7B, Figure S13B), but the extent of inhibition was greater in the *sng1* plants. Taken together, these results indicate that SNG1 inhibits root hair development and limits ABA‐induced inhibition of root hair elongation.

## DISCUSSION

The plant response to drought stress involves a wide range of physiological and biochemical processes to maintain water and ionic homeostasis and enhance survival chances. Recent research has identified several secondary metabolites involved in the regulation of drought stress (Kim et al., 2024; Muhammad Aslam et al., 2022), however, their precise roles in drought resistance are still obscure. We speculated that the sinapoyl ester may participate in drought resistance based on three lines of evidence. (i) Global metabolic analyses have shown that *sng1* plants contain slightly higher levels than WT plants of primary metabolites, such as soluble sugars (trehalose) and amino acids (proline), and secondary metabolites, such as anthocyanins and ascorbate, under normal light conditions (Kusano et al., 2011). The accumulation of these metabolites induced by UV‐B is also positively correlated with drought resistance (Jansen et al., 2022). (ii) Plants that overexpressed *WRKY50* contained higher levels of sinapic acid, SG and SM than WT plants and also showed enhanced drought resistance (Hussain et al., 2018). In addition, SCT/SNG2‐ deficient *sng2/sct* mutant plants contained higher levels of SG and were more resistant to drought stress than WT plants (Pieczynski et al., 2018). These results support a positive correlation between SG accumulation and drought resistance in Arabidopsis. (iii) Our previous investigation showed that sinapoyl ester metabolism affected ABA metabolism and signalling during seed germination and stomatal closure (Bi et al., 2017; Li et al., 2023), suggesting that ABA metabolism and signalling might be regulated by sinapoyl ester metabolism at other developmental stages. ABA has well‐documented roles in drought resistance (Hsu et al., 2021), and we therefore hypothesize that sinapoyl esters may also regulate plant drought resistance through the ABA signalling pathway. Sinapoyl esters are induced by UV‐B radiation and have a photoprotective role against UV radiation (Landry et al., 1995; Luo et al., 2017; Sheahan, 1996; Zhao et al., 2019), stomatal closure (Li et al., 2023) and S‐lignin synthesis (Cao et al., 2022; Huang et al., 2009). Here, we identified many metabolites function in plant response to drought, including sinapic acid, quercetin, etc in Arabidopsis by comparative metabolome analyses, and further research showed that both sinapic acid and quercetin relieved drought stress of Arabidopsis seedlings, however, sinapic acid exerted a far more pronounced effect (Figure 1, Figure S3), further study discovered an important role for sinapic acid in the response to drought stress in Arabidopsis, with SG‐accumulating *sng1* mutants showing increased resistance than WT (Figure 3). This result is also supported by a previous report showing higher SG levels and greater drought resistance in *AtWRKY50*‐overexpressing plants compared with WT plants (Hussain et al., 2018). The above‐mentioned data indicate that responses to UV‐B radiation and drought may share the sinapoyl ester metabolic pathway; as suggested by previous research based on gene expression, metabolic, physiological and morphological changes (Jansen et al., 2022; Poulson et al., 2006; Saenz‐de la et al., 2021). This study found that exogenous application of SA and quercetin both alleviated PEG6000‐mediated growth inhibition in Arabidopsis seedlings (Figure S3), whereas supplementation with SM produced no observable beneficial effects (Figure S2H,I). SM is a malate‐conjugated sinapoyl derivative stored in leaf tissue; the conversion of SG to SM is catalyzed by SNG1. Our previous research demonstrated that exogenous malate application restored stomatal aperture in *sng1* mutants to wild‐type levels (Bi et al., 2017). Taken together, we hypothesize that SM might affect drought responses in Arabidopsis, potentially *via* the modulation of stomatal closure. Quercetin is an antioxidant that is involved in several plant physiological processes, such as seed germination, pollen growth and photosynthesis and quercetin also induces ABA signalling regulation (Singh et al., 2021), how quercetin functions in ABA‐induced response to drought stress needs further studies to address.

Stomatal conductance and CO_2_ assimilation rate are tightly coupled under steady‐state conditions, with stomatal conductance limiting assimilation rate *via* CO_2_ supply (Sakoda et al., 2020; Toh et al., 2021). In Arabidopsis, reduced stomatal conductance under drought suppresses CO_2_ assimilation and subsequent growth, creating a trade‐off between drought tolerance and biomass accumulation (Ding et al., 2023; Drake et al., 2013). In this study, we found that the *sng1* mutant plants were smaller than the WT (Figures 2B, 4A and 5C, Figure S7A,B) and exhibited lower stomatal conductance (Figure 2E), which restricted CO_2_ assimilation. Meanwhile, the smaller plant size of the *sng1* mutant also reduced leaf water loss. We further determined that the *sng1* mutant had a slower water loss rate than WT (Figure 2C). This phenomenon is mainly attributed to the thicker cuticular wax accumulated on the leaf surface (Figure 4D,E) and the smaller stomatal aperture in the *sng1* mutant compared with WT (Figure 4F,G). In addition, our study revealed that SNG1 participates in regulating stomatal aperture in Arabidopsis but exerts no influence on the integrity of PSII (Figure S7C,D). Plant growth is modulated by multiple factors including CO_2_ assimilation, photosynthate metabolism, and water transpiration and absorption, which are closely associated with plant drought tolerance. Therefore, although SNG1 plays a vital role in coordinating plant growth and drought resistance in Arabidopsis, our experimental data indicate that SNG1 confers drought tolerance in Arabidopsis through diverse processes, with stomatal aperture and cuticular wax accumulation being the primary determinants.

One counterintuitive observation is that *SNG1*‐overexpressing Arabidopsis lines exhibited comparable SG levels to WT plants, yet the overexpressors were significantly more sensitive to drought stress than WT; we speculate that excessive expression of *SNG1* may couple with other signal transduction cascades to modulate drought responses, ultimately leading to drought‐related phenotypes distinct from WT. A similar regulatory pattern has been reported for AtSCPL1, a paralog of SNG1 in Arabidopsis: AtSCPL1 acts as a ubiquitination substrate of AtAIRP5/GARU and mediates drought tolerance in an ABA‐dependent manner (Cho et al., 2022). Here, we aimed to clarify the role of SNG1 in drought resistance by characterizing the *sng1* mutant using transcriptomic, physiological and morphological approaches.

Our transcriptome analysis suggested that wax biosynthesis and cuticle synthesis were enhanced in *sng1* plants (Figures 3D and 4D,E, and Tables 5A and 6A), with several wax biosynthetic‐ and cuticle synthesis‐related genes upregulated in *sng1* plants, including *ECERIFERUM 1* (*CER1*), *WAX ESTER SYNTHASE/DIACYLGLYCEROL ACYLTRANSFERASE 1* (*WSD1*) and *3‐KETOACYL‐COA SYNTHASE 1* (*KCS1*) (Figure S9). The increased expression of these genes observed in *sng1* mutants is consistent with the enhanced wax deposition and cuticle synthesis observed by SEM, cuticle permeability assays and GC–MS analysis (Figure 4B–E). The strong cuticular integrity of *sng1* plants may limit leaf water loss, contribute to the observed increase in leaf surface temperature (Figures 3B and 4A and Figure S10), and promote increased survival of *sng1* mutants under drought stress (Figure 2D). These findings suggest that SNG1 suppresses wax deposition and cuticle synthesis, thereby increasing leaf cuticular water loss and partially decreasing drought resistance.

In addition to water loss through the leaf cuticle, water is also lost through the stomata (Chen et al., 2020), and this pathway was also affected in *sng1* mutants. We identified several stomatal movement‐related DEGs regulated by SNG1 in our transcriptome analysis, such as *OPEN STOMATA 1* (*OST1*), *GUARD CELL HYDROGEN PEROXIDE‐RESISTANT 1* (*GHR1*) and *CHLORIDE CHANNEL a* (*CLCa*) (Figure S9), consistent with the smaller stomatal apertures observed in the *sng1* mutants (Figures 4F,G and 6A–C). These results indicate that SNG1 negatively regulates stomatal closure, consistent with our previous results (Li et al., 2023). Malate is considered to function as both an osmolyte and a signalling component in stomatal movement (Mimata et al., 2022). Due to the lack of suitable methods to detect malate concentration in guard cells, the malate content was quantitatively analysed in leaves and the lower epidermis of leaves. No differences in malate content were observed among leaves of WT, *sng1* and *fah1* (Figure 4H), consistent with our previous results (Li et al., 2023). However, the malate content in the lower epidermis of *sng1* and *fah1* was lower than that in WT (Figure 4H), indicating that the change in malate level in *sng1* and *fah1* may play a role in regulating stomatal aperture. Stomatal closure in response to exogenous ABA application was increased in *sng1* mutants compared to WT (Figure 6A–C), and similar results were observed in cotyledon opening assays (Figure 6D), seedling growth (Figure 6E,F), leaf senescence (Figure 6G,H) and root hair elongation (Figure 7B). These results suggest that the *sng1* mutant, which contained no SM and high levels of SG, showed enhanced ABA sensitivity.

Consistent with the results discussed above, we also observed higher ABA levels in *sng1* mutants compared to WT plants under well‐watered and drought stress conditions (Figure S12C), which partially explains the enhanced ABA responses in seedling growth (Figure 6E,F), leaf senescence (Figure 6G,H, Figure S8A,B), and root hair length (Figure S13B) by ABA accumulation in *sng1* mutants. The accumulation of ABA observed in *sng1* mutants may be related to increased expression of *NINE‐CIS‐EPOXYCAROTENOID DIOXYGENASE 3* (*NCED3*) and decreased expression of *CYP707A2*, which are key genes in ABA biosynthesis and catabolism, respectively (Chen et al., 2020). Likewise, the expression levels of ABA‐response genes, such as *RESPONSE TO DEHYDRATION 29A* (*RD29A*), *COLD‐REGULATED 15A* (*COR15A*) and *COR47*, were also higher in *sng1* compared to WT under normal and/or dehydration conditions (Figure S9). We also observed reduced cytokinin levels (*t*Z and *t*ZR) in rosette leaves of the *sng1* mutant (Figure S12D,E), which might also contribute to the observed increase in drought resistance, as previously documented for cytokinin‐related mutants (Nishiyama et al., 2011). The crosstalk between sinapoyl ester metabolism and ABA signalling, as well as cytokinin signalling, will require further investigation.

We observed intense dark‐purple colouration and higher anthocyanins levels in *sng1* mutants compared to WT plants after dehydration stress in agar (Figure 5A,B, Figure S11A) and under drought stress conditions in soil (Figure 5C–F, Figure S11B,C). Enhanced anthocyanin accumulation in *sng1* plants under drought stress may have been related to the observed higher expression of *MYB DOMAIN PROTEIN 75*/*PRODUCTION OF ANTHOCYANIN PIGMENT 1* (*MYB75/PAP1*), *DIHYDROFLAVONOL 4‐REDUCTASE* (*DFR*), *CYTOCHROME P450 75B1* (*CYP75B1*), *FLAVANONE 3‐HYDROXYLASE* (*F3H*) and *F3'H* genes (Tables S5 and 6B, Figure S9). We hypothesize that SG accumulation in *sng1* plants may redirect metabolic flux and induce the accumulation of other sinapoyl‐related downstream derivatives such as sinapoylated anthocyanins, 1,2‐di‐O‐sinapoyl‐β‐glucose and other sinapoyl esters (Milkowski & Strack, 2010; Santos‐Filho et al., 2012). Alternatively, accumulation of SG may exert negative feedback on sinapoyl metabolism enzymes, redirecting flux away from the sinapoyl pathway and toward other pathways (such as anthocyanin biosynthesis) that use common upstream precursors, such as phenylalanine. Similar results were observed in *kin10/kin11* mutant plants with downregulated *SnRK1* expression, which exhibited accumulation of SG and anthocyanin pigments but not SM (Wang et al., 2021). A positive correlation between SG levels and anthocyanin levels was also observed in the *fah1* mutant, deficient in accumulation of SG, SM compounds and anthocyanin pigments (Maruta et al., 2014). In summary, we hypothesize that accumulation of SG promotes anthocyanin biosynthesis by regulating and/or redirecting metabolic fluxes or by suppressing *SnRK1α/KIN12* expression, thus promoting the expression of *PAP1, DFR*, *CYP75B1*, *F3H* and *F3'H*. The accumulation of anthocyanins in *sng1* mutants may enhance their ROS scavenging capacity and thus increase drought resistance (Nakabayashi et al., 2014). ROS levels and ROS scavenging capacity of *sng1* plants require further investigation. These findings suggest that SNG1 may suppress anthocyanin accumulation, thereby decreasing ROS scavenging capacity and drought resistance.

In later developmental stages, old rosette leaves of *sng1* plants became yellow and died earlier than those of the WT (Figure 5C,G,H; Figure S7B,D). This observation was supported by the increased *SENESCENCE‐ASSOCIATED GENE 13* (*SAG13*) expression observed in *sng1* mutants (Figure S9). ABA‐induced senescence was also more pronounced in *sng1* mutants than in the WT (Figure 6G,H). These results indicate that SNG1 may negatively affect leaf senescence by suppressing ABA signalling. Leaf senescence positively regulates drought resistance by promoting the movement of limited water and nutrients from old leaves to young leaves to enable their survival (Munne‐Bosch & Alegre, 2004; Zhao et al., 2016). We therefore hypothesized that SG accumulation in *sng1* mutants promotes leaf senescence in an ABA‐dependent manner to enhance drought resistance and that SNG1 suppresses senescence in older leaves, thereby decreasing drought resistance.

In addition to shoot phenotypic changes, we also observed longer and more abundant root hairs in the *sng1* mutant compared to WT plants (Figure 7A), suggesting that *sng1* plants may have a stronger capacity for water and nutrient uptake (Comas et al., 2013). Many plant hormones, such as ABA, auxin and ethylene participate in the regulation of root hair development (Carbonnel et al., 2020; Li, Gao, et al., 2020; Lombardo & Lamattina, 2018; Villaecija‐Aguilar et al., 2021), and the detailed mechanisms by which plant hormones affect root development in *sng1* plants require further investigation. Nonetheless, our results suggest that SNG1 and sinapoyl esters (such as SG) may regulate root hair development through crosstalk with these plant hormone pathways.

A detailed characterization of additional genotypes with altered SG and SM levels, such as the *fah1* and *sng2*/*sct* mutants (Chapple et al., 1992; Fraser & Chapple, 2011; Pieczynski et al., 2018), would be very useful to clarify the positive function of sinapoyl esters in drought resistance. In this study, the sinapoyl esters (including SG and SM) production‐deficient mutant *fah1* was also found to have similar enhanced drought tolerance to *sng1* mutant plants (Figure 2A–D), while the *sng1* mutant showed reduced SM levels and increased SG accumulation (Lehfeldt et al., 2000). Therefore, we speculate that the reduced SM in *sng1* and *fah1* mutant plants may play a key role in enhancing plant drought tolerance. Although we did not investigate the drought resistance phenotypes of other sinapoyl ester mutants in this study, previous reports have shown that *sng2*/*sct* mutants are more tolerant to drought than WT (Pieczynski et al., 2018), thus supporting our hypothesis. A recent study found that at high sucrose concentrations, SnRK2.9 negatively modulates the stability and enzymatic activity of FAH1, suggesting the regulatory mechanisms employed by plants to modulate the anthocyanin response triggered by sucrose (Dong et al., 2025), these changes may be related to the function of FAH1 in drought stress. The detailed mechanisms and specific active components await further investigation.

Overall, based on metabolomic analysis of key ABA signaling mutants, sinapic acid was screened out as an important metabolite regulating drought resistance of Arabidopsis, followed by functional characterization of the SNG1; we hypothesize that the accumulation of SG or its downstream derivatives in *sng1* plants may enhance drought resistance by promoting cuticle synthesis, anthocyanin accumulation, leaf senescence, stomatal closure, ABA response and root hair development (Figure 7C). Furthermore, processes including cuticle synthesis, anthocyanin accumulation, leaf senescence, stomatal closure and root hair development are all regulated by ABA, further demonstrating that sinapic acid acts as a pivotal metabolite mediating ABA‐governed drought tolerance in Arabidopsis. There may be unidentified sensing components (receptors) or signal transduction mechanisms regulating these processes and, through them, plant adaptation to drought stress (Muro‐Villanueva et al., 2019) (Figure 7C).

## MATERIALS AND METHODS

### Plant materials, growth conditions and drought resistance

Seeds of *sng1‐1* (*sng1*) (Lehfeldt et al., 2000) and *fah1‐2* (*fah1*) (König et al., 2014) in the Col‐0 background were kindly provided by Clint Chapple (Purdue University, USA), and Col‐0 was used as the WT. Seeds of WT, *fah1* and *sng1* were incubated at 4°C in the dark for 3 d. After cold stratification, the seeds were sown on agar germination medium (GM) and grown in a growth chamber (16‐h light/8‐h dark cycle, 80 μmol m^−2^ s^−1^ photon flux density, 22°C). To compare the difference in drought resistance between *sng1* and WT plants, 2‐week‐old agar‐grown seedlings of the two genotypes were transferred side‐by‐side to a tray filled with water‐saturated soil (21 × 30 × 5 cm in width, length and depth). Thirty seedlings of each genotype were planted in each tray. For the first 2 days after transfer, the trays were covered with plastic film to maintain air humidity and promote rooting of the young seedlings. One week after transfer, the soil was irrigated to saturation again, and then water was withheld for the next 2 weeks. Trays were re‐watered when more than 15 plants of one genotype in one tray exhibited wilting leaves and stem bases. Three days after re‐watering, survival rates in all trays were assessed (Nishiyama et al., 2011).

### Metabolome profiling

All seeds were disinfected and stored at 4°C for 3 days to break dormancy in the dark. At that time, the seeds were still placed on 1.2% agar (m/v) containing 1/2MS medium. The photoperiod for incubating the plate at 22 ± 2°C is a 16‐h light/8‐h dark cycle, 80 μmol m^−2^ s^−1^ photon flux density. After 4 days of growth, transfer to a culture medium containing 20% PEG6000 or 1 μM ABA and 1/2MS was used as a control. After 9 days, the seedlings were extracted for metabolites for metabolome profiling analyses.

The metabolite samples in the Arabidopsis seedlings were prepared according to the reported method (ant et al., 2013). Liquid chromatograph mass spectrometer/mass spectrometer (LC–MS/MS) was used to measure metabolites using a timsTOF Pro system (BRUKER, Germany). The conditions were as follows: InfinityLab Poroshell 120 EC‐C18 (2.1 × 100 mm, 1.9 μm); capillary temperature, 220°C; aux gas flow rate, 10 L/min; eluent rate, 0.3 ml/min for 70 min. Metabolites were ascertained by automatic comparison to retention time, ion feature and tandem mass‐spectrometry fragmentation pattern (Dehaven et al., 2010). Following log2 transformation for profiling data and imputation of missing values, we performed univariate analysis (*t*‐test) to identify differential metabolites between control and treatment (*P* < 0.05 and FC >1.2 or <0.83). Differential metabolites were annotated using the KEGG database. Principal components analysis (PCA) and clustering heat maps were performed at MetaboAnalyst 5.0.

### Metabolomics analyses

The metabolomics analyses method was according to a previously reported study (Shu et al., 2023), partial least squares discriminant analysis (PLS‐DA) was used to distinguish the different metabolites among the groups. Variable importance of projection (VIP) values were determined using the OPLS‐DA model. Differential metabolites with VIP values greater than 1.0 and *P*‐values less than 0.05 were selected.

### Detached leaf water loss and RNA‐seq and transcriptome analysis

Twenty‐four‐day‐old soil‐grown seedlings were used to measure leaf relative water content (RWC). The rosette leaves of *sng1* and WT plants were removed and dried under our laboratory conditions. Weights of rosette leaves were recorded from 0 to 8 h at 1‐h intervals (FW_t_). When the dehydration treatment finished 8 h later, the rosette leaves were immersed in 40 ml distilled water in 50‐ml tubes at room temperature with shaking. When the rosette leaves had absorbed water and become fully saturated (~5 h), surface water was removed by briefly blotting with tissue paper, and the weight of the samples was recorded (turgid weight, TW). The leaf samples were then dried at 65°C for 2 days to obtain the dry weight (DW). RWC was calculated with the following equation:

$$\mathrm{RWC}\;\left(\%\right)=\left[\left({\mathrm{FW}}_{t}-\mathrm{DW}\right)/\left(\mathrm{TW}-\mathrm{DW}\right)\right]\times 100.$$

Samples for RNA‐seq were collected at the same time as samples for RWC measurement; rosette leaves were dehydrated and collected after 0 and 4 h of dehydration under the conditions described above. Total RNA was extracted from three biological replicates of each treatment and genotype using the TRIzol Reagent kit (Thermo Fisher Scientific). Detailed RNA‐seq and data analysis processes have been described previously (Love et al., 2014), and the resulting RNA‐seq data are available at the Gene Expression Omnibus (https://www.ncbi.nlm.nih.gov/geo/) under accession number PRJNA895105.

### Leaf surface temperature, TB staining and Chl leaching assays and observation of leaf surface waxes

To compare differences in leaf surface temperature between *sng1* and WT plants, 2‐week‐old agar‐grown seedlings of the two genotypes were transferred side‐by‐side into trays (21 × 30 × 5 cm in width, length and depth) filled with water‐saturated soil. Sixty seedlings of each genotype were planted in each tray at a high density to avoid the effect of soil temperature on leaf surface temperature. A thermal camera system (FLIR‐530; FLIR Optoelectronic Technology, USA) was used to measure leaf surface temperature of 24‐day‐old seedlings (14 days on agar and 10 days in soil). The same sets of 24‐day‐old *sng1* mutant and WT plants were used for the TB staining and Chl leaching assays following the detailed processes described in previous studies (Li, Nguyen, et al., 2020; Tanaka et al., 2004). Epicuticular wax was observed by SEM (Quanta 250, FEI) as described previously (Li, Nguyen, et al., 2020).

### Anthocyanin content and Chl measurements

For the agar‐grown seedlings, plates containing 14‐day‐old seedlings were opened and dehydrated on a laboratory bench for 1 h. The plates were then sealed again and grown in the same growth chamber for another 4 days. For the soil‐grown seedlings, the process was the same as that described for the drought resistance experiments. Rosette leaves were harvested after water had been withheld for 14 to 16 days. To measure anthocyanin levels, seedlings or rosette leaf samples were freeze‐dried (LGJ‐12D freeze drier; Beijing Sihuan Technology) for 48 h, and DWs were recorded. Leaf anthocyanin levels were measured according to a previously described method (Ito et al., 2015; Li, Nguyen, et al., 2020). Absorbance of the anthocyanin extracts was measured at 530 nm (A_530_) using a microplate reader (Epoch Microplate Spectrophotometer; BioTek Instruments, Inc).

For the ABA‐induced senescence assay, 10‐day‐old agar‐grown seedlings were transferred to new agar plates containing 0, 0.5, 1, or 2 μM ABA and grown for 12 days. After measuring the FW, samples were immersed in 10× extraction solution (95% acetone: ethanol = 2:1, v/v) at 4°C overnight in the dark. The absorbance of the resulting extracts was measured at 645 nm (A_645_) and 663 nm (A_663_). The Chl concentration of each solution was then calculated as follows: Chl concentration = [(20.2 × A_645_) + (8.02 × A_663_)]/FW.

### Assays of cotyledon opening, growth inhibition and stomatal closure in response to ABA

Seeds of the WT and the *sng1* mutant were sown on GM plates supplemented with 0, 0.5, 1 or 2 μM ABA and incubated at 4°C in the dark for 2 days. The plates were then transferred to a growth chamber, and assays of cotyledon opening and growth inhibition were performed as described in detail previously (Li, Nguyen, et al., 2020). The stomatal closure assay was also performed as described previously (Li et al., 2023). In brief, the fourth leaves were detached from 24‐day‐old WT and *sng1* mutant plants, and epidermal strips were prepared. The strips were first preincubated in 2‐(N‐morpholino) ethanesulfonic acid (MES)‐KCl buffer for 2 h under strong light, then transferred to fresh MES‐KCl buffer with different concentrations of ABA and incubated for another 2 h. Stomatal aperture sizes were measured after photographing the strips under a light microscope.

### Measurement of endogenous plant hormone contents

To measure the endogenous levels of plant hormones in *sng1* and WT plants under well‐watered and drought‐stressed conditions, 2‐week‐old agar‐grown seedlings of the two genotypes were transferred side‐by‐side to trays as described above. One week after transfer, the soil was irrigated to saturation, and water was then withheld for 10 days to obtain 31‐day‐old drought‐stressed seedlings. Well‐watered *sng1* and WT plants were grown at the same time with normal irrigation. Rosette leaves of the well‐watered and drought‐stressed plants were harvested and freeze‐dried (LGJ‐12D freeze drier/lyophilizer; Beijing Sihuan Qihang Technology, Beijing) for 48 h, and dry weights (DWs) were then recorded. The detailed extraction and purification processes were described previously (Simura et al., 2018), and labelled internal standards (IS) were added to the extraction solution.

ABA, tZ and tZR were quantified by ultra‐high‐performance liquid chromatography–tandem mass spectrometry (UPLC‐MS/MS). Purified samples were analysed using a Xevo TQ‐XS system (Waters, USA) equipped with an ESI ion source. Chromatographic separation was performed with an Acquity UPLC HSS T3 column (2.1 × 100 mm, 1.8 μm) maintained at 40°C. The mobile phase was composed of solvent A (0.1% formic acid, water) and solvent B (methanol), and the flow rate was set to 0.3 ml min^−1^. The linear gradient was as follows: 0–2 min, 2% B; 2–10 min, to 80% B; 10–12 min, 80% B; 12–13 min, to 2% B; 13–15 min, 2% B. The autosampler temperature was set to 4°C, and the sample injection volume was 10 μl. The MS data were collected in negative ion mode and multiple reaction monitoring (MRM) mode. The precursor and fragment ions were ABA (m/z 263.16–153.01), tZ (m/z 220.03–136.10), and tZR (m/z 352.2–136.02). Data analysis was performed using the spectrometer software (MassLynx v.4.2).

### qRT‐PCR

RNA was extracted with the TRIzol reagent (Thermo Fisher Scientific) and cleaned with an RNA Cleanup kit (Qiagen). Before use, the RNA was treated with DNase I (Sigma‐Aldrich) to remove contaminating DNA. HiScript qRT Supermix for qPCR (+gDNA wiper) (R123‐01; Vazyme Biotech, Nanjing, China) was used for reverse transcription and cDNA synthesis from the same RNA samples used for RNA‐seq analysis. qRT‐PCR was performed using SYBR Green (TaKaRa Bio Inc., Kusatsu, Shiga, Japan) on a qTOWER 2.2 Real‐Time PCR Thermal Cycler (Analytik Jena AG, Germany). The *UBQ10* gene was used as the reference gene. The 2^−ΔΔCt^ method was used to calculate the relative gene expression levels (Livak & Schmittgen, 2001). Gene‐specific primers are listed in Table S7. Statistical analysis was performed using two‐tailed Student's *t*‐tests based on biologically replicated data.

### Analysis of sinapoyl esters

Determination of sinapoyl esters is as described in our previous study (Bi et al., 2017) with slight modifications. Rosette leaves of the well‐watered and drought‐stressed plants were harvested and freeze‐dried (LGJ‐12D freeze drier/lyophilizer; Beijing Sihuan Qihang Technology, Beijing) for 48 h, and dry weights (DWs) were then recorded. Rosette leaves of Arabidopsis were ground into powder in liquid nitrogen and extracted for 30 min at 50°C with 50% methanol containing 25 μM chrysin as an internal standard. The vortexed sample should be centrifuged at 13000 × ***g*** for 10 min. then the supernatant was filtered through a 13 mm mini spike 0.2 μm Nylon filter. Sinapoyl ester levels were determined by high‐performance liquid chromatography (HPLC, Waters e2695 HPLC System with 2998 PDA Detector). The sample was resolved on a SunFire 5 μm C18 Column in 0.1% formic acid (A) with an increasing concentration gradient of acetonitrile containing 0.1% formic acid (B) at a constant rate of 0.5 ml min^−1^: 0 min, 5% B; 0–10 min, 20% B; 10–20 min, 40% B; 20–35 min, 95% B. UV absorption was monitored at 320 nm using a multiple‐wavelength photodiode array detector (Waters). Peaks were identified and quantified using commercially available standard substances.

### Wax content measurement

Waxes were extracted from rosette leaves of 4‐week‐old Arabidopsis plants grown as described above by dipping in CHCl_3_ for 1 min (Yadav et al., 2014). After that, 20 μl (500 μg/ml) of tetracosane and heptadecanoic acid standard solution was added to the sample extract as an internal standard and dried under nitrogen blowing. 100 μL of BSTFA (with 1% TMCS) and 100 μl of pyridine were implanted into the dried sample and incubated at 70°C for 60 min to harvest derivatization. The derivatized product was removed from the incubator and cooled to room temperature, followed by evaporating the excess derivatized reagent with a slow nitrogen flow at 60°C. Derivatized samples were evaporated to dryness and dissolved in hexane.

GC–MS analysis was performed using a Xevo TQ‐GC gas chromatography‐tandem mass spectrometer (Waters, United States) equipped with an HP‐5MS column (30 m × 0.25 mm i.d. × 0.25 μm film thickness, J&W Scientific, USA) to analyse the derivatized products of the samples. The GC–MS conditions were set as follows: helium was used as the carrier gas at a flow rate of 1 ml/min. Split injection mode was applied with a split ratio of 10:1, and the injection volume was 2 μl. The oven temperature was initially set at 60°C with a 1.5‐min delay, then increased to 200°C at 10°C/min, further increased to 320°C at 5°C/min and held at 320°C for 15 min. All samples were analysed in the scan mode over a mass range of m/z 40 to 650. The inlet, ion source and transfer line temperatures were 280°C, 230°C and 280°C, respectively.

### Lignin content measurement

Determination of lignin is as described in a previous study (Zhao et al., 2023) with slight modifications. The stems from 6‐week Arabidopsis plants were harvested, lyophilized and ball milled. The obtained fine powders were extracted with 70% (v/v) ethanol at 65°C for 3 h and then centrifuged to obtain pellets. The pellets were resuspended in 70% (v/v) ethanol, and the extraction was repeated twice. The obtained pellets were then extracted with chloroform/methanol (1:1, v/v) at room temperature for 1 h, as was the ethanol extraction. The process was repeated 3 times, and the pellets were finally extracted with acetone at room temperature overnight. After centrifugation, the residues were collected and dried at room temperature to obtain extractive‐free cell wall residues. Approximately 10 mg of extractant‐free residues was weighed and placed into a glass vial, followed by the addition of a freshly prepared reaction mixture composed of 2.5% boron trifluoride etherate and 10% ethanethiol in dioxane (v/v). The vials were purged with nitrogen gas before being sealed, then placed in a 95°C heat block for 4 h with intermittent shaking. After cooling, sequentially add an appropriate amount of 0.4 M sodium bicarbonate, 2 ml of water and 1 ml of dichloromethane (containing 1 mg/ml methyl heptadecanoate), and adjust the pH to between 3 and 4. The vials were vortexed for 1 min and centrifuged at 1000 × ***g*** for 5 min to separate the phases. The organic phase was collected and dried in a heat block overnight at 50°C. Subsequently, 0.5 ml of methylene chloride was added to resuspend the dried sample, and 50 μl of the sample was transferred to a new centrifuge tube and dried under the same conditions. The sample was then derivatized by adding 50 μl of pyridine and 50 μl of N‐methyl‐N‐(trimethylsilyl) trifluoroacetamide; after incubation for at least 5 h at room temperature, 1 μl of the reaction product was analysed by GC–MS.

GC–MS was conducted on gas chromatography tandem mass spectrometry Xevo TQ‐GC (Waters, United States). The analyses used were as previously described (Li et al., 2023). In brief, the GC–MS was equipped with a DB‐5 column (30 m × 0.25 mm × 0.25 μm, Agilent Technologies). The oven initial temperature was maintained at 130°C for 3 min and then increased to 250°C at 3°C/min, and finally maintained at 250°C for 5 min. The injector temperature was maintained at 250°C with a constant flow rate of 1.0 ml/min of helium. The inlet was operated in pulsed splitless injection mode. The GC–MS electron impact source was operated in scan mode with the MS source temperature at 280°C and MS Quad at 150°C.

### Determination of malate concentration

The determination method of malate concentration, as described in (Dong et al., 2018), malate was extracted from rosette leaves or the lower epidermis of Arabidopsis that had grown for 4 weeks. Each sample was ground into powder in liquid nitrogen and was extracted for 30 min at 4°C with distilled water. Malic acid was quantified by ultra‐high‐performance liquid chromatography–tandem mass spectrometry (UPLC‐MS/MS). Samples were analysed using a Xevo TQ‐XS system (Waters, USA) equipped with an ESI ion source. The data were collected in negative ion mode, multiple reactions monitoring (MRM) mode. Precursor and fragment ions are: m/z 132.97‐115.1. Chromatographic separation was conducted by an ACQUITY UPLC HSS T3 column (2.1 × 100 mm, 1.8 μm) maintained at 40°C. The mobile phase, composed of solvent A (0.1% formic acid, water) and solvent B (methanol), flow rate was set as 0.2 ml min^−1^. The linear gradient system was set as follows: 0–1.5 min, 0% B; 1.5–3 min, to 5% B; 3–4 min, 20% B; 4–5 min, to 95% B; 5–7 min, 95% B; 7.5–10 min, 0% B. The autosampler temperature was set to 4°C, and the sample injection volume was 5 μl.

### Stems lignin staining

Hand sections (with a thickness of 100–120 μm) were prepared from the same position of stems of 6‐week‐old WT, *sng1* and *fah1* plants. For phloroglucinol‐HCl staining, sections were immersed in 1% phloroglucinol (0.1 g phloroglucinol in 10 ml 95% EtOH) for 3–5 min; afterwards, sections were gently rinsed with water and acidified with 20% HCl (Li et al., 2022). For Mäule staining, 0.5% potassium permanganate solution was added to the sections for staining; the solution was washed out and then distilled water was added to rinse out the potassium permanganate solution; finally, 3% HCl was added, and it was incubated for 3–5 min until the deep brown colour was discharged from the sections. After pipetting out all the HCl solution, concentrated ammonium hydroxide solution was added immediately (Qin et al., 2020). Finally, the sections were examined by an inverted microscope.

### Statistical analysis

Data are presented as the mean ± standard deviation (SD) or mean ± standard error (SE) from at least three independent experiments (*n* ≥ 3). Different letters above the error bars indicate statistically significant differences (*P* < 0.05; analysis of variance followed by Tukey's honestly significant difference test) among different genotypes and treatments, which were calculated using SPSS statistical software (IBM, USA).

## AUTHOR CONTRIBUTIONS

YM, WL and KL conceived and designed the experiments. JJ, YY, MZ, XL, ZW, ZL, MX, HL and YW performed the experiments. YM, WL, KL, SZ and FY analysed the data. L‐SPT, JG, KJ, SZ, JJ and FY contributed reagents/materials/analytical tools. YM, WL and KL wrote the paper. L‐SPT and JB revised the manuscript.

## CONFLICT OF INTEREST

The authors have no conflicts of interest to declare.

## Supporting information


**Table S1.** Counts and fold changes for comparative metabolome analysis of WT seedlings under normal growth and PEG6000‐ and ABA‐treated conditions.
**Table S2.** Counts and fold changes for comparative metabolome analysis of WT, *snrk‐triple* and *pyl‐quadruple* seedlings.
**Table S3.** The Fragments per kilo base per million mapped reads (FPKM) of all genes from rosette leaves of *sng1* and wild‐type (WT) plants under well‐watered and 4 h dehydration conditions.
**Table S4.** Fold changes (FCs) for comparing gene expression from rosette leaves of sng1 and wild‐type (WT) plants under well‐watered and 4 h dehydration conditions.
**Table S5.** The characterization of upregulated genes of ‘*sng1*‐C vs. WT‐C’ and ‘*sng1*‐D vs. WT‐D’ comparisons.
**Table S6.** Enrichment analysis of upregulated genes of ‘*sng1*‐C vs. WT‐C′ and n*sng1*‐D vs. WT‐D′ comparisons.
**Table S7.** List of primers used for qRT‐PCR.

**Figure S1.** The WT seedlings were inhibited by 20% PEG6000.
**Figure S2.** Comparative metabolome analysis of WT plants under ABA and PEG–treated conditions.
**Figure S3.** Phenotypic analysis of Arabidopsis seedlings treated with sinapic acid and quercetin under PEG6000‐induced stress.
**Figure S4.** The expression level of *FAH1*, *BRT1* and *SNG1*.
**Figure S5.** Soil moisture contents after stopping watering.
**Figure S6.** *SNG1* overexpression plants are more sensitive to drought stress than WT and *sng1* plants.
**Figure S7.** Fresh weight and the maximum quantum yield of PSII photochemistry (Fv/Fm) of WT, *sng1* and *fah1* plants.
**Figure S8.** The lignin contents and composition in WT, *sng1* and *fah1* plants.
**Figure S9.** qRT‐PCR quantification of gene expression to validate RNA‐seq results.
**Figure S10.** Leaf surface temperatures of WT and *sng1* plants after stopping water for 7 days (28‐day‐old plants).
**Figure S11.** Anthocyanins accumulation in seedlings of *sng1* and WT.
**Figure S12.** The *sng1* leaves were sensitive to ABA.
**Figure S13.** Root hair density and relative root hair length of *sng1* and WT in response to ABA.

## Data Availability

All relevant data can be found within the manuscript and its Supporting Materials.
